# A review of visual sustained attention: neural mechanisms and computational models

**DOI:** 10.7717/peerj.15351

**Published:** 2023-06-13

**Authors:** Huimin Huang, Rui Li, Junsong Zhang

**Affiliations:** 1 National Engineering Research Center for E-learning, Central China Normal University, Wuhan, Hubei, China; 2 Brain Cognition and Intelligent Computing Lab, Department of Artificial Intelligence, School of Informatics, Xiamen University, Xiamen, Fujian, China

**Keywords:** Sustained attention, Computational models, Neural mechanisms, Evaluation, Neural pathways

## Abstract

Sustained attention is one of the basic abilities of humans to maintain concentration on relevant information while ignoring irrelevant information over extended periods. The purpose of the review is to provide insight into how to integrate neural mechanisms of sustained attention with computational models to facilitate research and application. Although many studies have assessed attention, the evaluation of humans’ sustained attention is not sufficiently comprehensive. Hence, this study provides a current review on both neural mechanisms and computational models of visual sustained attention. We first review models, measurements, and neural mechanisms of sustained attention and propose plausible neural pathways for visual sustained attention. Next, we analyze and compare the different computational models of sustained attention that the previous reviews have not systematically summarized. We then provide computational models for automatically detecting vigilance states and evaluation of sustained attention. Finally, we outline possible future trends in the research field of sustained attention.

## Introduction

Attention acts as a gate for information flow in the brain (Cohen, 2014), allowing the brain to concentrate on processing continuous information. The term “attention” comes from the Latin “attentus”, which is the past participle of attendere, which means “to heed” (Itti & Baldi, 2005). Although the word existed in Roman times, little scientific research was conducted on it until philosophers and pioneering psychologists paid attention to it. Attention research has primarily interested specialists in psychology because attention is linked to many mental disorders. Human beings with attention-deficit disorders such as dyslexia (Walda et al., 2021), traumatic brain injury (Carroll et al., 2020), depression (Vaughn-Coaxum et al., 2021), and attention-deficit/hyperactivity disorder (ADHD) (Mansour et al., 2021) will have difficulty concentrating. As one of the fundamental cognitive abilities, attention has been the subject of research by experts in various research fields, including philosophy, physiology, neuropsychology, clinical, education, and computer science (Cohen, Sparling-Cohen & O’Donnell, 1993). However, interdisciplinary research is often considered overly difficult. Interdisciplinary challenges in the field of sustained attention remain unresolved, given the differences in conceptual definitions and research methods across interdisciplinary fields.

Current studies rarely mention sustained attention, which is the basis of other attention types (for example, selective attention) and plays an irreplaceable role in humans’ daily lives. For instance, selective attention relates to focus and determines which information is given priority over others, while sustained attention refers to long-term focus and is typically related to vigilance (Cohen, 2014). Sustained attention is characterized by the ability to detect rare and unpredictable signals over a long period of time (Munir, Cornish & Wilding, 2000). Sustained attention or vigilance refers to the ability to maintain a consistent behavioral response to task-related stimuli during continuous and repetitive activity (Robertson et al., 1997). The key to the above definitions is that sustained attention is focused on the performance of a single task over a period of time. The essential difference between sustained and transient attention is that transient attention is a transient event-related state, while sustained attention is a sustained block-level state that shows attentional fluctuation over a long activity duration (Li et al., 2019).

Human senses can process an enormous amount of information. However, the brain cannot maintain attention over long periods of time to process the constant influx of information from the environment. One of the most important characteristics of visual sustained attention is the ability to make target-present or target-absent decisions rapidly and accurately (Warm, 1984). The first relevant investigation into the visual sustained attention phenomenon is Mackworth’s mission of military personnel surveillance radar during World War II (Mackworth, 1950). At present, the world has entered the era of informatization and digital multimedia. Diverse and complicated information unrelated to the current task can easily divert attention. Sustained attention can ensure a more lasting focus on a task (Chen & Wu, 2015). However, sustained attention is affected by mental fatigue and is frequently diverted to irrelevant information. Humans are especially prone to fall into a state of mental fatigue when tasks require them to maintain a high level of attention for a long time. Furthermore, fatigue often reduces task performance by affecting vigilance (Thompson et al., 2020a; Thompson et al., 2020b). The ability to detect relevant information decreases as the time required to maintain sustained attention increases, a phenomenon known as “vigilance decrement”. Humans in a low-vigilance state tend to experience mind wandering (Jin, Borst & van Vugt, 2020). Humans with low sustained attention will be unable to complete tasks and may even exhibit symptoms of attentional disorders such as ADHD. To evaluate the level of sustained attention, several studies in the past made use of machine learning combined with neuroimaging technology. Aggarwal et al. (2021) assessed attention levels for students in a massive online open course learning environment using EEG signals. Shoeibi, Ghassemi & Rajendra Acharya (2022) used a new deep learning method to build an ADHD intelligent detection model to assess resting-state functional magnetic resonance imaging (rs-fMRI) data.

Although sustained attention is vital to humans’ daily lives, several challenges hinder its exploration in research fields: (1) Previous researchers have often used self-reflection questionnaires, electrocardiograms, eye movements, and electroencephalograms (EEG) to evaluate the sustained attention of humans. However, no complete and comprehensive routine assessment of sustained attention exists due to the different research methods involved in different disciplines. (2) There have been many attempts to develop guidelines for human learning and work based on neuroscience findings. Therefore, neural mechanisms of sustained attention must be introduced to enhance our understanding of neuroscience-based methods for increasing task efficiency. (3) Sustained attention evaluation for the large number of existing attention-deficit patients usually requires clinical diagnosis by clinicians, which is time-consuming and labor-intensive. A large quantity of data has been produced by advanced neuroimaging techniques and an increasing number of researchers have utilized computational models to evaluate sustained attention. Therefore, machine learning, a relatively new advanced computing method, is uniquely suited for processing large-scale data generated by neuroimaging techniques (Jo, Nho & Saykin, 2019).

Accordingly, the aims of the present article are as follows: (1) to provide a comprehensive understanding of what sustained attention is and how it can be measured, we review theoretical models in ‘Sustained attention: state-of-the-art models’ and introduce paradigms of psychological experiments in ‘Paradigms for psychological experiments on sustained attention’. (2) To explore how sustained attention can effectively promote task performance, we review studies on the neural mechanisms of sustained attention and propose possible visual pathways in ‘Neural mechanisms’. (3) To give a panorama of computational models for the automatic diagnosis of sustained attention, we review various computational models for measuring attention in ‘Computational models’. (4) To illustrate possible directions in the research field of sustained attention, we outline its applications and future trends in ‘Conclusions’.

## Survey Methodology

In our daily lives, we are surrounded by constant visual information, but our visual processing capacity is limited. Human access to information is dominated by visual information (Treichler, 1967). A large number of neurons are dedicated to analyzing human visual information, which makes vision an indispensable sense (Kaewkhaw et al., 2015). Sustained attention is crucial when a visual task calls for prolonged attention and ongoing stimulus monitoring (Loetscher et al., 2019). Sustained attention was accompanied by the flow of top-down visual spatial attention signals in human parietal and occipital topographic cortical areas (Lauritzen et al., 2009). However, the visual pathways of sustained attention have not been fully clarified in previous studies. Therefore, in this review, we attempt to cover the neural mechanisms of visual sustained attention and computational models applied to study attention, especially sustained attention. First, we provide theoretical models and experimental paradigms related to sustained attention assessment for the convenience of readers without cognitive science backgrounds. We further explore the neural mechanisms and plausible neural circuits of sustained attention. Finally, we review various computational models of attention.

We used Google Scholar as well as PubMed databases to search for relevant articles in our publication survey. First, we used the search terms “visual attention”, “visual pathway”, “sustained attention”,“ attention pathway”, “machine learning”, “deep learning”, “sustained attention assessment”, and “neural mechanisms of sustained attention”. We then expanded our search, including “attention disorders”, “computational models”, “attention assessment”, “neural mechanisms of attention”, and “visual pathways of attention”. Second, we expanded our examination of computational models applied to attention given that there are fewer computational models for sustained attention. We rigorously searched for publications focusing on the application of computational models to attention research. Then, the search term was specified as the name of the computational model described earlier. We consistently excluded irrelevant studies throughout the review process. For publications that met our criteria, we deeply reviewed their computational approaches to sustained attention and categorized them into different computational models. In addition, we compared articles on different computational models with other relevant studies we found. We removed publications that did not match our approach.

As we attempted to capture all available studies of the neural mechanisms of sustained attention, the year of our collection of publications is from 1948 to the present. Additionally, to understand machine learning models of sustained attention, we collected research published after July 2008 based on search criteria.

## Results

We found four basic research topics that received sustained attention. These included theoretical models of sustained attention, experimental paradigms of sustained attention, neural mechanisms and computational models of sustained attention. We reviewed these accordingly.

### Sustained attention: state-of-the-art models

**Figure 1:**
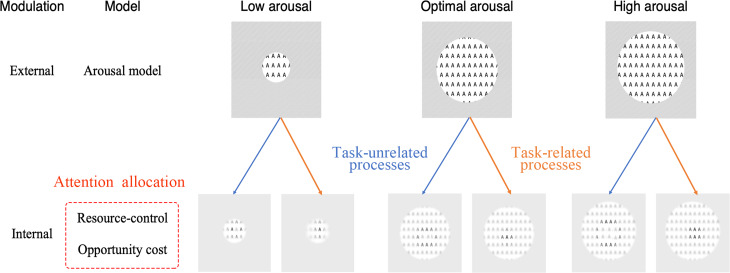
Models of sustained attention (Chun, Golomb & Turk-Browne, 2011). Task performance is influenced by various factors, including arousal, attentional allocation, and information processing. Arousal is the level of physiological and psychological activation, which can be determined by various factors, including emotions, motivation, and environmental stimuli. Attentional allocation is influenced by the intrinsic cost of control, motivation, and the degree of arousal. The circles represent the degree of arousal, and the larger the circle, the higher the degree of arousal. Insufficient attentional state in low arousal states affects task performance. The optimal arousal ensures sufficient attention for the task. Excessive arousal states can lead to low task performance due to distraction. Different degrees of arousal are controlled by internal cognition, such as resource-control and opportunity cost, to regulate the proportion of attentional resources. Higher internal controls can handle multitasking or more difficult tasks (the more As in bold), and lower internal controls can only handle single or simple tasks. Blue arrows indicate process of task-unrelated distractors. Red arrows indicate process of task-related targets.

The purpose of the sustained attention survey is to explain the individual’s internal fluctuations during the task as well as his or her overall ability to maintain the task (Esterman & Rothlein, 2019). Moreover, the success of maintaining sustained attention is dependent on modulating both external and internal distractions. Therefore, Chun, Golomb & Turk-Browne (2011) classified attention as external modulation and internal modulation based on whether the attention goal was sensory stimulation (external) or cognitive control processes (internal) (see Fig. 1). Under this taxonomy, sustained attention includes maintaining both external and internal attentional focus as well as persistence over a period of time.

Several theoretical models were proposed to illustrate sustained attention from various perspectives (Hancock, 1989; Hancock & Warm, 2003; Blotenberg & Schmidt-Atzert, 2019; Esterman & Rothlein, 2019). The arousal model modulates sustained attention through the locus coeruleus (LC), affecting the external signal-to-noise ratio and internal information-processing ability. This model suggests that the state of arousal is closely related to our perception and sensory stimulation, as external stimuli automatically capture attention and trigger bottom-up processing. Conversely, top-down control occurs when attention is voluntarily allocated to the internal mind. Moreover, optimal physiological arousal is essential for sustained attention. Arousal is also not a static state. Even if humans are not tired, their arousal levels fluctuate as they become interested, afraid, or surprised. Arousal is mainly regulated by noradrenaline, a neurotransmitter secreted by the locus coeruleus of the midbrain, according to molecular neuroscience (Aston-Jones & Cohen, 2005). The LC-norepinephrine system receives projections from the orbitofrontal cortex and the anterior cingulate gyrus. By enhancing the activity of specific neurons or inhibiting the activities of unrelated neurons, the LC-norepinephrine system optimizes individual behavior through arousal in regular and persistent activities (Lenartowicz, Simpson & Cohen, 2013). In addition, the acetylcholine system in the dorsal pons and basal forebrain, the serotonin system in the raphe nucleus, the histaminergic systems in the tuberomammillary nucleus, and the orexigenic systems in the lateral hypothalamus all contribute to regulating arousal levels through cortical activation. Therefore, the arousal state involves a series of internal physiological changes related to external modulation *via* the activity of neurotransmitters to enhance task-related information-processing ability. Moreover, after the brain receives the stimulus signal, heart rate, electrophysiological activity, and pupil will change in response to the competition from participants’ responses to different sources of stimuli (Unsworth, Robison & Miller, 2018).

While the arousal model explains neural regulation of sustained attention from a neurophysiological perspective, it is difficult to explain the decrease in internal vigilance and the allocation of attention resources. Therefore, a resource-control model (Thomson, Besner & Smilek, 2015) and an opportunity-cost model (Kurzban et al., 2013) have been proposed. These models suggest that the level of intrinsic motivation during a specific task, and the ability to exert influence, diminishes over time (Fortenbaugh, De Gutis & Esterman, 2017). The resource-control model explains the decrease in vigilance over time by the tendency for attentional resource distraction, which is influenced by task difficulty and time course (Thomson, Besner & Smilek, 2015). Generally, increasing the difficulty and duration of a task requires more available attention resources to be used, which increases the demand for attention resource allocation (See et al., 1995). Attention resources are associated with the central executive attentional network (Gartenberg et al., 2018). The depletion of central executive network resources affects sustained attention, leading to errors in information perception and processing. Moreover, considering time-on-task performance, executive control decreases with increased mind wandering, resulting in more attention resources being devoted to mind wandering over time. From the perspective of alternative underload, mindlessness and goal habituation also cause a decline in vigilance (Helton & Russell, 2012). Several behavioral and neuroimaging studies support the resource-control model. A study based on fMRI found that several brain regions associated with vigilance, including the basal ganglia, the sensorimotor cortex, and a right-sided frontal-parietal attention network, were activated after the psychomotor vigilance test (Lim et al., 2010). Vigilance activates the thalamus, as well as the anterior and posterior cortex areas that are potentially related to norepinephrine during the attention network test (Fan et al., 2005). Additionally, sustained attention is related to the functional connections between the default network and the dorsal attention network (Esterman et al., 2017). However, the resource-control model is primarily based on visual modality studies, and it is still unclear whether it can be applied universally to other sensory modalities (Terashima et al., 2021).

Although the resource-control model provides a task-related explanation, it does not elaborate on the effect of subjective experience (mental effort) on task performance (Esterman & Rothlein, 2019). The opportunity-cost model was proposed to explain the decrease in vigilance based on the psychological representation of subjects (Kurzban et al., 2013). It focuses on the expected value of vigilance tasks rather than the proportion of attention resources consumed by mind wandering. The cost and benefit of “effort” in task performance are related to psychological representation. The motivation to devote attention to tasks depends on the effort and psychological expectation of the task execution. Manipulation of the model can explain the impact of psychological activities, such as intrinsic motivation, interest, reward, and stress, on time-on-task performance (Esterman et al., 2016). Attentional engagement and time-on-task performance fluctuations were associated with motivation (Brosowsky et al., 2020). Moreover, a large-scale brain attention network was selectively activated in response to the stimulus characteristics of the task (Long & Kuhl, 2018).

The studies mentioned above suggest that sustained attention models can distinguish multiple states of optimal attention due to external or internal modulation. Moreover, these studies reveal that attention, as a limited resource, can affect task performance over time. However, the neural mechanisms by which attention affects task performance are not fully understood (Esterman & Rothlein, 2019). Therefore, more studies are still needed to investigate the neural basis for theoretical models of sustained attention.

### Paradigms for psychological experiments on sustained attention

To explore the neural mechanisms of sustained attention, neuroimaging and electrophysiological methods are used to reveal the neural activity of basic cognitive processes in sustained attention. EEG and magnetoencephalography (MEG) have a high temporal resolution of submilliseconds, allowing them to detect rapid changes in electrophysiological responses. Functional magnetic resonance imaging (fMRI) uses endogenous blood oxygenation level-dependent (BOLD) contrast to map human brain activity. Moreover, rs-fMRI brain networks in sustained attention tasks can predict differences in individual performance. It can locate brain regions activated by different tasks or stimuli with a millimeter spatial resolution. While these neuroimaging techniques can effectively monitor potential neural activity, it is vital to design experimental paradigms that can genuinely evoke the neural activity associated with sustained attention. Therefore, many tasks, also called vigilance or sustained attention tasks, have been designed to monitor and evaluate sustained attention. In Table 1, we introduce several experimental paradigms commonly used to measure sustained attention.

Vigilance tasks are used to assess the capacity of sustained attention over long periods. Among the vigilance tasks, the Mackworth clock task (MCT) was a game changer in the 1940s (Mackworth, 1948). It was developed to assess the vigilance of radar technicians during World War II. MCT has been shown in many studies to decrease participant vigilance during tasks (Arsintescu, Mulligan & Flynn-Evans, 2017). Participants monitor the forward ticks of a clock hand and respond when the tick is twice the usual.

Although MCT has been replicated in various studies, the continuous performance test (CPT) is the most reliable and well-recognized approach for the clinical evaluation of vigilance (Arsintescu et al., 2019). The CPT is now applied as a kind of neuropsychological test to assess humans’ inattentiveness, impulsivity, and vigilance. Moreover, the CPT has been proven to be sensitive to sustained attention. The CPT has evolved in the last century into different versions. The Conners’ CPT (Conners & Sitarenios, 2011), also called the nonX CPT, is the most widely accepted version of the CPT. The Conners’ CPT is mainly used to assess vigilance task performance and reaction inhibition. The participants needed to press the space bar when they saw nontarget stimuli (non-X), while they needed to withhold a response to the target letter X. The stimuli occurred at 1-, 2-, or 4-s interstimulus intervals (ISIs) during the Conners’ CPT. All Conners’ tests took 14 min to administer.

**Table 1:** Paradigms in visual sustained attention.

**Paradigms**	**Participants**	**Presentation**	**Blocks**	**Sub-blocks**	**ISIs**	**Respondents**	**Duration**
CCPT	≥ 8 years	‘A–X’ letters	6	three 20-trials	–	‘non-X’	12 min
CPT-II	≥ 8 years	‘A–X’ letters	6	three 20-trials	1, 2, or 4 s	‘non-X’	14 min
CPT-III	≥ 8 years	‘A–X’ letters	6	three 20-trials	1∼4 s	‘non-X’	14 min
TOVA	4–80 years	two grey squares	2	125 trials	–	bottom square	21 min
ARCPT	≥13	‘A–X’ letters	10	100 trials	adaptive	‘A-X’ sequence	25 min
SART	14–77 years	digits	3	18 trials	1 s	digit “3”	4-6 min
PVT	≥ 14 years	Red millisecond counter	14	112 trials	2∼10 s	Red counter	13 min
GradCPT	≥ 16 years	Pictures of cities and mountains	1	497 trials	–	city	10 min
MCT	adults	A white face and a black pointer	4	100 discrete steps	$ \frac{3}{4} $∼3 min	double hops	2 h
Stroop	≥ 8 years	Color-word	3	96 trials	1 s	Red, yellow, blue, green	20 min

**Notes.**

ISI inter stimulus intervals CCPT conners’ continuous performance test CPT continuous performance test TOVA test of variables of attention ARCPT adaptive rate continuous performance test SART sustained attention to response task AVT abbreviated vigilance task PVT psychomotor vigilance task gradCPT gradual-onset continuous performance task MCT Mackworth Clock Task

In addition to Conners’ CPTs, the test of variables of attention (TOVA) measures the ability to maintain attention with an additional auditory component. Compared with those in other CPTs, the reaction time measurement in TOVA is more accurate and sensitive. Each target (a square near the top edge) or nontarget (a square near the bottom edge) appeared on a computer screen for 100 ms, and participants were asked to press a spacebar if the presented stimulus was a target picture. Many experimental studies have shown that the TOVA is reliable and effective in evaluating ADHD. Most of the results show that TOVA is helpful in distinguishing subjects who have problems with attention lapses (Lin et al., 2021). The adaptive rate continuous performance test (ARCPT) differs from the CCPT and TOVA in that it measures sustained attention on a more demanding rapid information-processing task (Lohr, 1999). The ISIs of the ARCPT are adaptive and vary depending on the performance of the subjects. The initial ISI is set to 60 ms. If the response to the stimulus is correct, the ISI will decrease by 4 ms; otherwise, it will increase by 4 ms after the error. The changing ISIs enable participants to maintain an accuracy of 80% in the task.

Although the ISIs of the ARCPT allow participants to maintain a high accuracy level, it is difficult to solve the reaction errors caused by subjects’ boredom or mind wandering Cohen, Sparling-Cohen & O’Donnell (1993). Therefore, Manly & Robertson (2005) presented sustained attention to response tasks (SART) as a measurement of sustained attention. The SART is a go/no-go vigilance task used to measure sustained attention in short periods of time. The mechanically continuous response in the SART causes the participant to endogenously regulate attention. When the subjects saw the frequent stimuli (for example, digit ‘3’), they had to press the space-bar. However, when they saw the infrequent stimuli, they had to withhold response. Because of its conciseness, the SART has been utilized extensively in clinical practice research of sustained attention as well as in a variety of brain imaging studies (Scheinost et al., 2020). Furthermore, the SART has also been used as an additional vigilance test in some sleep studies.

In addition to the SART, the psychomotor vigilance test (PVT) is also related to the measurement of sustained attention in sleep research (Dinges & Powell, 1985). The PVT aims to assess changes in performance caused by decreased vigilance. Participants were instructed to maintain their fastest possible reaction time to a visual stimulus (typically, a milli-second counter) at random 1–9 s ISIs. It has been widely used in research on fatigue. The decline in performance during the PVT is primarily due to cognitive slowing and attention lapses Dinges & Kribbs (1991).

In the experimental paradigms described above, external cues caused by abrupt onsets and offsets of stimuli cannot be easily eliminated. The gradual-onset continuous performance task (gradCPT), which uses fade-in and fade-out for stimulus presentation, can better clarify the behavioral and neural correlates of visual sustained attention (Esterman et al., 2013). Participants were required to press buttons for city scene images (90% of trials displaying city scenes, 10% mountain scenes). Each scene image gradually transitioned, occurring over 800 ms. Several other paradigms are also used to test broader attention-related problems (Munnik et al., 2020).

In conclusion, the ultimate goal of different experimental paradigms is to evoke cognitive processes associated with sustained attention during tasks. Moreover, researchers who use the same paradigm tend to frame their questions similarly.

### Neural mechanisms

Exploring the neural mechanisms of sustained attention helps in understanding the human psychophysiological process during tasks. Traditional research has mainly focused on the activities of specific brain regions during sustained attention (Sarter, Givens & Bruno, 2001; Sonuga-Barke & Castellanos, 2007). An increasing number of researchers are beginning to recognize that brain areas involved in sustained attention are not limited to specific areas (Klimesch, 2012; Pamplona et al., 2020). In general, the neural mechanisms of sustained attention include visual, auditory, and other somatosensory pathways (Clayton, Yeung & Cohen Kadosh, 2015; Helfrich et al., 2018). However, visual attention is probably more widely known among all cortical systems than auditory and somatosensory attention. Studies have shown that many brain areas, primarily the occipital, parietal, temporal, and frontal eye fields, are involved in the human visual attention system (Saygin & Sereno, 2008; Offen et al., 2010; van et al., 2022).

Regarding brain areas involved in sustained attention, Clayton, Yeung & Cohen Kadosh (2015) addressed this issue by proposing an oscillatory model in which the posterior medial frontal cortex (pMFC), medial prefrontal cortex (mPFC), posterior cingulate cortex (PCC), and lateral prefrontal cortex (LPFC) are primarily involved in sustained attention. Langner & Eickhoff (2013) used functional neuroimaging to identify 14 clusters that were consistently activated across various tasks involved in sustained attention. These clusters are primarily found in the frontal cortex, cingulate cortices, and subcortical structures (see Fig. S1 ).

#### Sustained attention and the frontal lobe

The frontal lobe is a section of the brain that covers the front part of the cerebral cortex. It is usually regarded as the executive control center of the brain (Luria, 1973). These executive functions consist of a number of individual capacities, such as inhibition, goal-directed behavior, and self-monitoring (Oliveira et al., 2012). These individual capacities control and regulate the process of sustained attention. The frontal lobe mainly comprises the premotor area, primary motor area, and prefrontal lobe. The prefrontal cortex is located in the anterior region of the frontal lobe. It is linked to higher-order processing abilities such as attention, working memory, language, and executive function (Raver & Blair, 2016). Frontal lobe system damage often affects sustained attention, resulting in lapses in sustained attention and attention-executive disorders (Esterman et al., 2013). Moreover, it was found that patients with injuries in some areas of the prefrontal cortex performed abnormally in the implementation of sustained attention tasks, whereas they performed normally in other cognitive ability tests (Sarter, Givens & Bruno, 2001; Langner & Eickhoff, 2013). The frontal cortex has rich functional connections with the posterior brain system as well as several subcortical systems, including the limbic system, midbrain reticular system, and thalamic structure.

Many neurophysiology studies have found increased activation in the frontal cortex during a vigilance task (see Table S1). In addition, Han, Lee & Choi (2019) proposed that theta and alpha-band (4–12 Hz) EEG activities in the frontal cortex were essential for sustained attention and goal-related behaviors. In particular, EEG activity of the CPT state shows the dominance of effective connections going from the prefrontal cortex toward the parietal lobe at 4 Hz (Francisco-Vicencio et al., 2022). Moreover, sustained attentional preparation can be indexed by the deployment of a centrally distributed event-related potential (ERP), named the contingent negative variation (CNV) (Segalowitz, Dywan & Unsal, 1997; Kropp et al., 2001). CNV and P3 within the frontal cortex appear to be good candidates to investigate different mechanisms supporting sustained attention and prediction abilities (Thillay et al., 2015).

Damage to the whole frontal cortex or attention-related brain region (such as, pMFC, mPFC) affects sustained attention function, manifesting as behavioral disturbances or functional abnormalities in an individual. For example, rs-fMRI study found that frontal functional disconnection may underlie the pathogenesis responsible for defective vigilance/sustained attention (Tu et al., 2020). In addition, increased functional connectivity in the right frontoparietal network might reflect excessive cognitive fatigue in patients with traumatic brain injury (TBI) (Shumskaya et al, 2012).

Deficits in sustained attention are the most common disorder caused by frontal cortex damage (Wilkins, Shallice & McCarthy, 1987). Many studies have shown that abnormal neuron development in the frontal lobe may cause sustained attention disorders or even hyperactivity disorders (Rubia et al., 2019). In addition to developmental disorders, stroke and brain tumors in the frontal cortex can also lead to sustained attention deficits (Torres et al., 2021). Closed head injuries caused by external impact or sudden violent exercise can also impair sustained attention (Parasuraman, Mutter & Molloy, 1991). Furthermore, sustained attention decreases with aging because of frontal lobe degeneration over time (Mitko et al., 2019). Patients with deficits in sustained attention often suffer from ADHD, epilepsy, depression, intellectual disability, and other complex neuropsychiatric problems as well (Malkovsky et al., 2012). Among these symptoms, ADHD is a representative disorder of deficits in sustained attention (Kass, Wallace & Vodanovich, 2003). It has been linked to inattention, impulsivity, and negative affect (Barkley, Knouse & Murphy, 2011). Patients with ADHD have difficulty maintaining focus and vigilance for extended periods of time, leading to poor academic performance, career mistakes, and even operator-related train/car accidents (Fortenbaugh, De Gutis & Esterman, 2017; Zeller, 2022).

#### Sustained attention and the cingulate cortex

The cingulate cortex consists of two distinct systems: (1) a posterior system that receives input from dorsal stream areas and projects to some cortical systems, the thalamus, and (2) an anterior system that receives signals from the thalamus and frontal-parietal lobes and projects to limbic structures (Jafari, Malayeri & Rostami, 2015). Some studies have revealed that the cingulate cortex participates in sustained attention tasks (see Table S2).

The anterior cingulate cortex (ACC) receives inputs from the lateral frontal cortex and the posterior parietal cortex. In addition, it compactly connects with the basal ganglia (BG). The anterior cingulate cortex, which is part of the limbic system, receives inputs from the thalamus and neocortex and has large projections to the nucleus accumbens and amygdala. Many experts have found that the anterior cingulate cortex plays a vital role in conflict monitoring (Jones et al., 2002). The ACC was found to be associated with cognitive impairment in rs-fMRI of sustained attention tasks (Loitfelder et al., 2012). Furthermore, some researchers have confirmed that the anterior cingulate cortex is constantly activated during tasks related to sustained attention (Fan et al., 2018). Subsequent studies using a series of Stroop tasks have suggested that strong reactions in the anterior cingulate cortex mediate attention and conflict resolution (Corlier et al., 2020) (see Table S2). Moreover, in healthy adults, better sustained attention was associated with more robust activation of the ACC during SART and gradCPT tasks (Esterman et al., 2013).

The posterior cingulate cortex (PCC) is considered to be a paralimbic cortical structure. It has rich projections to the frontal, parietal, and temporal cortex, as well as to subcortical systems such as the thalamic nucleus, pontine, and basal ganglia. Therefore, the PCC is thought to be involved in several cognitive activities, although its specific functions have not been clarified. The results of resting functional brain imaging revealed that the PCC is a critical node in the default mode network (DMN) and plays a vital role in attention regulation (Kral et al., 2019). It has been confirmed that the PCC is activated and has strong interactions with other parts of the DMN in both resting state and continuous working memory tasks (Lau, Leung & Zhang, 2020). Abnormalities of the DMN are frequently seen in neurological and psychiatric disorders such as ADHD, Alzheimer’s disease, schizophrenia, autism, and depression. Therefore, PCC has important clinical significance (Zhou et al., 2020).

The latest neuroimaging research found that there is a selective enhancement of oscillatory coupling between the ACC and the dorsal attention network (DAN) during attention tasks (Wong et al., 2022). Human single-neuron recordings during conflict tasks suggest that the dorsal ACC can be involved in attention-related performance monitoring (Fu et al., 2022). Resting-state functional connectivity within the DAN can predict individual performance in spatial attention tasks (Machner et al., 2022).

#### Sustained attention and subcortical structures

Subcortical structures are neural structures located deep in the brain that include the brainstem, midbrain, cerebellum, basal ganglia, thalamus, hypothalamus, and limbic nuclei. The hypothalamus and the reticular formation coordinate arousal through their vast array of projections to other brain regions (Rapoport et al., 1978). The basal ganglia and thalamic nucleus are responsible for processing gating information. They are closely related to attention and somatic movement (McAlonan, Cavanaugh & Wurtz, 2008). According to Fan et al. (2008), the basal ganglia appear to be central to executive regulation mechanisms, error monitoring, and sustained vigilance. Limbic nuclei include the amygdala, septal nucleus, and nucleus accumbens.

The anterior thalamic nuclei may serve as a site of integration between frontal areas and the hippocampus to regulate attentional processes (Nelson, 2021). Attention is also linked to the hippocampus, which is responsible for the storage, conversion, and orientation of long-term memory (Aly & Turk-Browne, 2016). Evidence from the reticular formation (Dietrich & Audiffren, 2011), thalamus (Rajab et al., 2014), and limbic structures (Wang et al., 2013b) suggests that exercise may help to facilitate attentional processes. The asymmetrical development of the right-lateralization of the frontal lobe and left-lateralization of the occipital lobe may affect ADHD severity (Chen et al., 2021). Furthermore, stereo-electroencephalography (SEEG) recordings provide direct evidence that the anterior nucleus of the thalamus modulates hippocampal gamma activity in attention and working memory tasks (Piper et al., 2022; Liu et al., 2021).

Based on the information above, attention is a byproduct of regulation from multiple brain regions rather than a strictly cortical phenomenon. Clinical studies have provided additional evidence that the nervous system contains the frontal lobe, cingulate cortex, and subcortical system, which play an appropriate role in sustained attention.

#### Neural pathways of visual sustained attention

Sustained visual attention is necessary for humans’ visual systems to have incredible perception and data processing capabilities. Many studies have been dedicated to exploring the neural pathways of sustained visual attention.

Dynamic causal modeling provides compelling evidence for the regulation of attention through the PFC ↔thalamic, ACC ↔thalamic, BG ↔thalamic, and PFC ↔BG pathways (Jagtap & Diwadkar, 2016). For example, the modulation of thalamic →PFC pathways is presumed to reflect ascending attention processes engaged by external sensory inputs of salient and novel stimuli. In comparison, modulation of frontal →thalamic pathways represents descending attention processes mediated by voluntary shifts of attention based on expectations of goals and rewards (Connor, Egeth & Yantis, 2004). Before reaching the cortex, visual information is filtered by the thalamus. The thalamus, the “gateway” to the cortex, comprises various subnuclei involved in attention gating (Brunia, 1993). The thalamus affects feedforward and feedback information transmission between the frontal, parietal and occipital cortex regions (Tokoro et al., 2015). Attention to stimuli suppresses the neuronal activity of the reticular nucleus over selected relay nuclei, and this disinhibition gates thalamocortical inputs (Conway, 2014). These functional effects appear to be mediated by anatomical connections between the thalamus (and specific thalamic nuclei) and regions of the frontal lobe, including the LPFC and the cingulate cortex. The PCC is closely linked to the thalamus (Leech & Sharp, 2014). It receives information from the visual cortex and sends it to the LPFC (Buschman & Kastner, 2015). Sustained attention responses also exist in the early visual cortex in the absence of visual stimuli (Silver, Ress & Heeger, 2007). Several elegant studies have found that the presupplementary motor area (pre-SMA) is the target of the BG (Akkal, Dum & Strick, 2007; Wiesendanger & Wiesendanger, 1985). The pre-SMA receives input from the cortex and delivers output to the thalamus. The BG organizes motivations that lead to the execution of goal-directed behaviors, for example, pushing a button. When the attention process in the ventral regions is goal-oriented, information from the visual cortex activates neural activity in the inferotemporal cortex (IT), which is followed by activation in the LPFC (Hommel et al., 2019). The anteromedial prefrontal cortex, ACC, anterior insula, and anterior thalamic nodes form the cingulo-opercular circuit, which is involved in distinguishing potential mismatches and conflicts (Williams, 2016).

**Figure 2:**
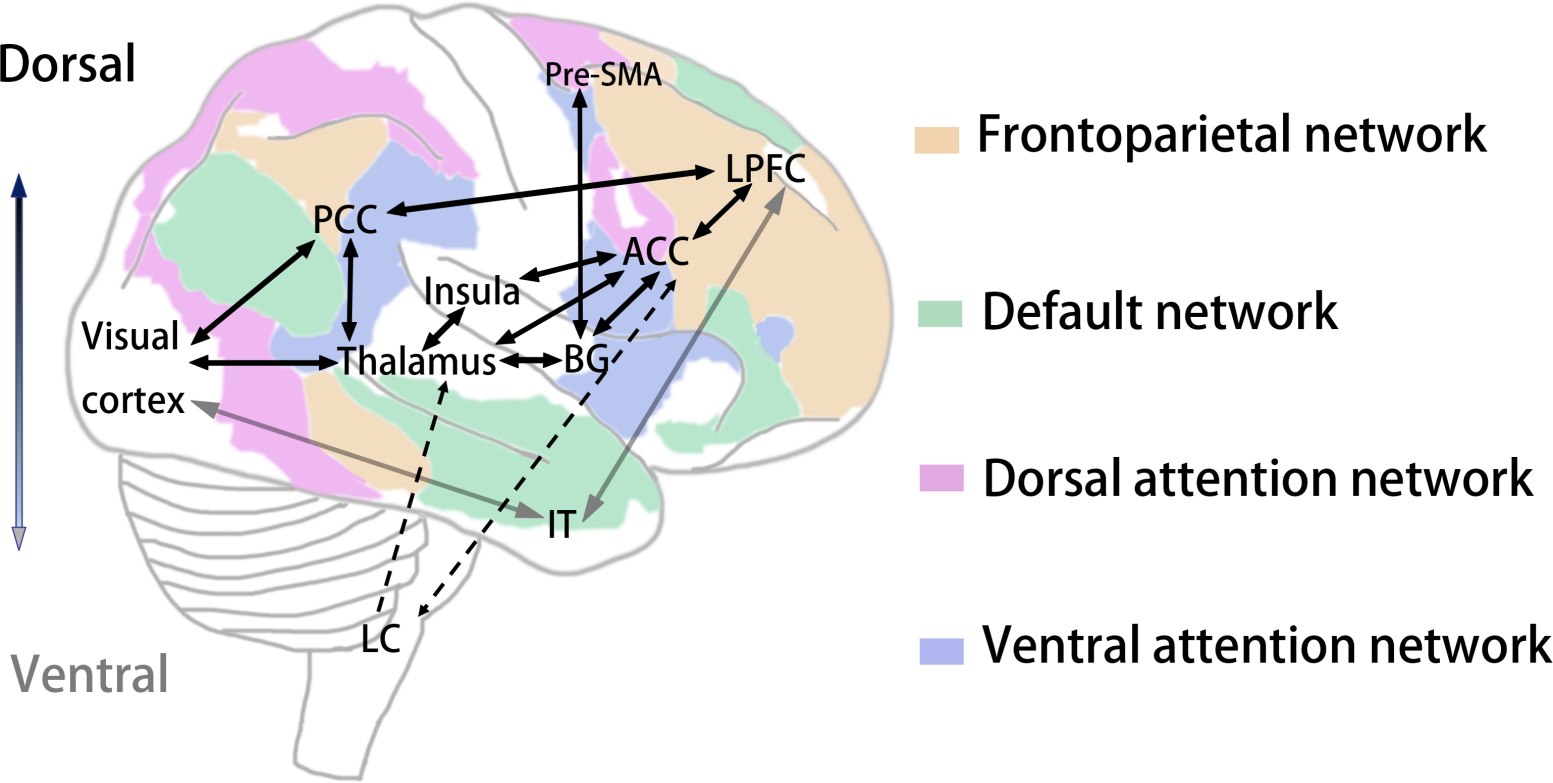
A plausible visual sustained attention pathway. From visual cortex, through LPFC, two major routes have been described, a dorsal pathway through subcortical structures (and related areas), and a ventral pathway through IT cortex. The visual attention dorsal pathway has been implicated in top-down attention at objects, features, or regions in space for sustained periods of time (the black solid line), while the ventral pathway represents bottom-up attention is transiently captured (the gray solid line) (Pinto et al., 2013; Conway, 2014). The brain network of visual sustained attention consists of sub-networks including cingulate cortex, LPFC, thalamus, insula, BG, and IT (Jagtap & Diwadkar, 2016). These sub-networks are responsible for functions including regulation, error monitoring or processing, and sustained vigilance. In addition to forebrain, the midbrain LC can also regulate sustained attention by secreting neurotransmitters (the black dashed line). LPFC, lateral prefrontal cortex; pre-SMA, pre-supplementary motor area; ACC, anterior cingulate cortex; PCC, posterior cingulate cortex; IT, inferotemporal; BG, basal ganglia; LC, locus-coeruleus.

In addition, the arousal model shows that the LC-norepinephrine system in the brainstem plays a critical role in the vigilance of sustained attention. Norepinephrine projections originating from the LC and ending in the thalamus mediate the attention process (Sarter, Givens & Bruno, 2001). Projections from the ACC to the LC-norepinephrine system indicate that the mPFC is involved in the regulation of arousal through low-frequency phase synchronization with the LPFC (Clayton, Yeung & Cohen Kadosh, 2015; Craigmyle, 2013). Based on the above literature, we propose possible pathways of sustained attention, as shown in Fig. 2.

In addition, functional brain networks play a crucial role in sustained attention. Recent research has posited that the visual processes of sustained attention emerge from an array of large-scale functional networks (Fortenbaugh et al., 2018). In largely independent lines of research, influential brain network models (Esterman et al., 2013) have suggested that optimal sustained attention requires cooperation among the task positive network (TPN), frontoparietal control network (FPN), ventral attention network (VAN), dorsal attention network (DAN), and DMN, as shown in Fig. 2. Intracranial electroencephalography (iEEG) in human subjects offers evidence that the DMN interacts negatively with both the DAN and salience network (SN) (Kucyi et al., 2020). Moreover, researchers who utilized BOLD of fMRI found that many attention-related brain networks, such as the DMN and DAN, were activated during the gradCPT task (Mitko et al., 2019). Better sustained attention is associated with stronger anticorrelations between the DAN and DMN (Chang et al., 2022). Furthermore, optimal sustained attention is less dependent on the DAN and more dependent on brain networks related to task automation, such as the DMN (Okabe, 2016). Therefore, characterizing both anatomic neural pathways and functional connectivity could allow for a more profound study and eventually provide a panorama of the neural mechanisms of sustained attention.

### Computational models

The theoretical models used to explain sustained attention are constantly evolving as cognitive science advances. The arousal and resource-control models mentioned above describe the complex relationship between multiple processing modules in sustained attention. However, descriptive models cannot quantitatively analyze the relationship between these modules. Moreover, descriptive models provide little information about how the processing modules in sustained attention change under specific conditions. It is difficult to give a precise measurement of sustained attention. Therefore, cognitive and computer scientists have introduced many computational models to quantitatively measure and evaluate sustained attention.

#### Biomathematical models

Early research on computational models was related to sleep loss and circadian rhythm. Researchers usually ask subjects to perform a vigilance task after sleep deprivation when sleep is insufficient or irregular. Then, the subject’s vigilance or sustained attention can be quantified by using a computational model that is based on task performance. A diverse set of vigilance tasks will cause a decline in performance as time-on-task increases (Davies & Parasuraman, 1982). These vigilance tasks proved that the reduction magnitude is influenced by various factors, including the event rate, signal probability, stimulus duration, stimulus modality, and others (Warm, Dember & Hancock, 1996; Greenlee & Hess, 2019). At present, sleep loss and fluctuations in circadian rhythms are used by researchers to explain the reasons for decreased vigilance (Walsh, 2014). There appear to be notable differences in the study of theories about sleep deprivation and vigilance reduction. However, studies have shown that insufficient sleep and reduced vigilance have the same effects on cognitive processing (Veksler & Gunzelmann, 2018). Kronauer, Forger & Jewett (1999) found that ambient light can affect vigilance by influencing the phase and amplitude of circadian pacemakers. Jewett & Kronauer (1999) proposed a circadian rhythm neurobehavioral performance and alertness (CNPA) model. Hursh et al. (2004) proposed a sleep activity, fatigue, and task effectiveness (SAFTE) model. These biomathematical models predict that an increase in sleep deprivation will lead to a continued decline in vigilance.

Therefore, early biomathematical models can only provide estimates of vigilance. They cannot predict potential changes in performance or cognitive processing, nor can they explain the mechanism of behavioral changes.

#### Integration models

Researchers integrated predictions of alertness levels, generated by biomathematical models, with information-processing methods in cognitive architecture model to produce precise predictions of sustained attention. Cognitive architecture model provides a unified information-processing framework that is based on decades of empirical evidence and psychological theories. Thus, the integration model not only helps in understanding the changes in human performance as vigilance declines but also explains the underlying neural mechanisms of sustained attention.

Anderson et al. (1998) proposed a cognitive architecture known as adaptive control of thought-rational (ACT-R). It has been used to provide a quantitative description of human performance in cognitive tasks. Gunzelmann et al. (2011) introduced the microlapse theory of fatigue (MTF), which integrated a biomathematical model with ACT-R to model different sustained attention tasks. MTF, as an instantiation of the computational model, describes the process of decreased vigilance. Jackson et al. (2013) improved Gunzelmann’s model by including a time-on-task component. Although Jackson’s model uses the same mechanism as the original model, it has an impact on sleep deprivation and circadian rhythm research. Gartenberg et al. (2018) proposed the microlapse theory of fatigue with replenishment (MTFR), a process model similar to MTF that supplements the mechanisms related to opportunistic rest periods and internal rewards.

These computational models, when combined with a specific cognitive framework, can achieve a strong fit between simulated and actual behavioral data to predict behavioral performance in sustained attention tasks.

#### Machine learning algorithms

With the advancement of modern science, neuroimaging technology is increasingly being used to evaluate sustained attention (Zhang et al., 2022). Previously, neuroimaging data were combined with classical statistical methods to construct a computational model of sustained attention. However, as research advances, neuroimaging technology will result in an explosive increase in data scale. Classical statistical methods cannot handle large quantities of neuroimaging data, and manually processing these complex neuroimaging data is time-consuming. Thus, computational models that can automatically and elegantly process massive quantities of neuroimaging data are urgently needed to meet the demands of state-of-the-art neuroimaging research. Machine learning, an advanced computing method, is uniquely suited to address these issues.

In traditional machine learning approaches, handcrafted features are commonly combined with support vector machines (SVMs), k-nearest neighbors (KNNs), and Bayesian networks. The classification accuracy is over 70% when solving a binary classification problem in most EEG-based studies (see Table 2).

**Table 2:** Studies of sustained attention with machine learning models.

**Author**	**Type of subjects**	**Number of subjects**	**Research method**	**Data sources**	**Average accuracy**	**Classification**
Cirett Galán & Beal (2012)	Healthy, age ≥ 18 years	*N* = 16 (8 males)	SVM	EEG	73%	Engagement and workload
Zhang et al. (2016)	Healthy	*N* = 15	SVM	fNIRS	80%	Three levels of attentional load
Yeo, Shen & Wilder-Smith (2009)	Healthy, age 20–25 years	*N* = 20 (10 males)	SVM	EEG	99.30%	Alert and drowsy state
Samaha, Sprague & Postle (2016)	Healthy, age 18–27 years	*N* = 8 (7 males)	SVM	EEG	95%	Different frequency bands
Batbat, Güven & Dolu (2019)	Healthy, age 18–25 years	*N* = 48 (28 males)	SVM	EEG	88.89%	Selective attention and divided attention
Öztoprak et al. (2017)	ADHD, non-ADHD	*N* = 25	SVM-RFE	EEG	100%	ADHD and non-ADHD
Miao & Zhang (2017)	ADHD age 11.19 ± 2.63 years. non-ADHD age 12.41 ± 3.28 years	ADHD (*N* = 82), non-ADHD (*N* = 72)	SVM	fMRI	92.16%	ADHD and non-ADHD
Ghassemi et al. (2010)	ADHD, non-ADHD, age 29.78 ± 6.15 years	ADHD (*N* = 10) (7 males), non-ADHD (*N* = 40) (19 males)	KNN	EEG	92%	ADHD and non-ADHD
Ghassemi et al. (2012)	ADHD, non-ADHD, age 29.78 ± 6.15 years	ADHD (*N* = 10) (7 males), non-ADHD (*N* = 40) (19 males)	KNN	ECG	96%	ADHD and non-ADHD
Hu, Sun & Ratcliffe (2016)	Healthy, age 20–25 years	*N* = 10 (7 males)	CFS+KNN	EEG	80.84%	Attention on high, neutral, low
Borji, Sihite & Itti (2012a); Borji, Sihite & Itti (2012b)	Healthy	–	KNN	Behavioral	–	Mapping from features to saccade locations
Chen et al. (2010)	Healthy, age 18–24 years	*N* = 28	KNN	ECG	98%	Attention and non-attention
Larue, Rakotonirainy & Pettitt (2010)	Healthy, age 22.6 ± 9.2 years	*N* = 40 (8 males)	Bayesian	Behavioral	72%	Vigilance decline
Weigard, Huang-Pollock & Brown (2016)	ADHD, age 9.96 ± 1.21 years. non-ADHD, age 10.18 ± 1.31 years	ADHD (*N* = 66) (39 males), non-ADHD (*N* = 66) (36 males)	Bayesian	Behavioral	75%	Predicting human visual attention
Pang et al. (2008)	Healthy	–	Bayesian	Behavioral	–	Evaluating the consequences
Luo et al. (2020)	Healthy	–	Bayesian	Behavioral	–	Predicting human visual attention
Borji, Sihite & Itti (2012a); Borji, Sihite & Itti (2012b)	Healthy	*N* = 10	Bayesian	Behavioral	–	Predicting human visual attention

**Notes.**

SVM support vector machines EEG electroencephalogram fNIRS functional near-infrared spectroscopy ADHD attention-deficit/hyperactivity disorder SVM-RFE support vector machines recursive feature elimination fMRI functional magnetic resonance imaging KNN k-nearest neighbors ECG electrocardiography CFS correlation-based feature selection

SVM is a supervised learning model for classification and regression in machine learning. It performs well when recognizing small samples with high-dimensional data. By using the SVM model, some studies focused on assessing the attentional state in healthy people. Yeo, Shen & Wilder-Smith (2009) tested the usefulness of SVM in identifying or distinguishing between alert and drowsy EEG patterns. Cirett Galán & Beal (2012) used SVM to estimate sustained attention and cognitive workload while students were solving a series of math problems. Zhang et al. (2016) used SVM to detect sustained attention load based on passive brain-computer interface signals from functional near-infrared spectroscopy (fNIRS); Samaha, Sprague & Postle (2016) used SVM to assess whether the spatial selectivity of neural responses can be recovered from the topography of alpha-band oscillations during spatial attention. Batbat, Güven & Dolu (2019) achieved high accuracy by combining EEG data from visual, auditory, and auditory-visual tasks and using an SVM with a linear kernel to classify different attentional states. Moreover, SVM is regarded as a relatively fast classifier. It is practically suitable for cases where the number of features is greater than the number of instances. However, SVM is difficult to implement in large-scale samples and to address multilabel classification problems.

The KNN algorithm, unlike the SVM algorithm, is an instance-based learning method. The KNN classifier is very simple and intuitive. High accuracy was achieved in classifying clinical patients and nonclinical participants using a combination of features with a KNN classifier. There are studies that have distinguished between different attentional states. For example, Chen et al. (2010) classified sustained attention phases using electrocardiograms (ECG) with KNN. Borji, Sihite & Itti (2011) used KNN to predict human attention in the training set by implementing a nonlinear mapping from eye position to task state. Hu, Sun & Ratcliffe (2016) proposed a classification method that combines correlation-based feature selection (CFS) and a KNN algorithm to identify attentional states during the learning process. However, KNN depends heavily on training data. The complexity of KNN increases dramatically as the number of features increases.

The Bayesian model is more adaptable than the two other methods. It is a type of probabilistic graph model that uses Bayesian inference to compute the probability. The Bayesian model is fast and has been used for attentional classification problems by many researchers, such as Larue, Rakotonirainy & Pettitt (2010). In their study, the participants’ reaction time during the SART was used to detect vigilance decline in real time using a Bayesian model. They quantified the effect of monotony on overall performance. In addition, a Bayesian model can also be used to predict the likelihood of humans typically focusing on a scene (Pang et al., 2008). Luo et al. (2020) developed a hybrid incremental dynamic Bayesian network and constructed a visual focus detection method based on fusing drivers’ head and eye movement data. Borji, Sihite & Itti (2012a); Borji, Sihite & Itti (2012b) used Bayesian networks to estimate the attentional state of subjects while performing a task (for example, playing video games) and mapped the state to an eye position. In addition to the classifiers mentioned above, principal component analysis (Arruda et al., 2007) (PCA), artificial neural networks (ANNs) (Dowman & Ben-Avraham, 2008), linear discriminant analysis (LDA) (Ghassemi et al., 2009), K-Means (Gurudath & Bryan Riley, 2014), and other methods have been used to solve attention-related classification problems.

Classic machine learning algorithms require experts to design elegant, handcrafted features. However, deep learning can learn feature representations from datasets automatically. To interpret data, deep learning builds a deep neural network that mimics the neural mechanisms of the human brain (Zaharchuk et al., 2018). As data acquisition technology advances in scientific research, computer-aided data analysis based on deep learning will become more widely accepted. Therefore, we reviewed existing deep learning methods for attentional state classification (see Table 3), including convolutional neural networks (CNNs) and recurrent neural networks (RNNs).

**Table 3:** Studies of sustained attention with machine learning models.

**Author**	**Type of subjects**	**Number of subjects**	**Research method**	**Data sources**	**Average accuracy**	**Classification**
Borhani et al. (2018)	Healthy, age 21–24 years	*N* = 38 (27 males)	CNN	EEG	73%	2 classes attentional state
Hosseini & Guo (2019)	Healthy, age 25,31 years	*N* = 2 (1 male)	CNN	EEG	91.78%	Focusing state and mind wandering
Ho et al. (2019)	Healthy	*N* = 16 (8 males)	CNN	fNIRS	65.43%	3 classes mental workload
Khullar et al. (2021)	ADHD, non-ADHD, age 7–21 years	ADHD (*N* = 285), non-ADHD (*N* = 491)	2D CNN–LSTM	fMRI	98.12%	ADHD and non-ADHD
Zou et al. (2017)	ADHD, non-ADHD, age 7–21 years	ADHD (*N* = 286), non-ADHD (*N* = 340)	CNN	MRI	69.15%	ADHD and non-ADHD
Mao et al (2019)	ADHD, non-ADHD, age 7–21 years	ADHD (*N* = 285), non-ADHD (*N* = 491)	4-D CNN	fMRI	71.3%	ADHD and non-ADHD
Chen et al. (2019)	ADHD age 10.44 ± 0.75 years, non-ADHD age 10.92 ± 0.69 years	ADHD (*N* = 50) (41 males). non-ADHD (*N* = 57) (53 males)	CNN	EEG	90.29%	ADHD and non-ADHD
Fouladvand et al. (2018)	ADHD, age 13–20 years	*N* = 11,624	LSTM	–	84%	Temporal medication features
Phan et al. (2018)	Healthy	*N* = 20	RNN	EEG	82.50%	5 sleep stages
Liu & Liu (2017)	Healthy	*N* = 27	RNN	EEG	74.50%	Arousal and valence
Huve, Takahashi & Hashimoto (2018)	Healthy	–	RNN	fNIRS	63%	Driving under clear weather and driving under rainy weather
Rasyid & Djamal (2019)	Healthy	–	RNN	EEG	89.73%	Emotion and attention
Moinnereau et al. (2018)	Healthy	–	RNN	EEG	83.2%	3 auditory stimuli
Ming et al. (2018)	Healthy	*N* = 16	RRN	EEG	89.3%	2 classes attentional state
Kuang & He (2014)	ADHD	*N* = 222	DBN	fMRI	35.19%	4 classes attentional state
Kuang et al. (2014)	ADHD	*N* = 94	DBN	fMRI	61.90%	4 classes attentional state
Vahid et al. (2019)	ADHD, non-ADHD	*N* = 144	EEGNet	EEG	83%	ADHD and non-ADHD
Hao & Yin (2015)	ADHD, non-ADHD	*N* = 873	–	fMRI	64.70%	ADHD and non-ADHD
Dubreuil-Vall, Ruffini & Camprodon (2019)	ADHD age 43.85 ± 14.78 years. non-ADHD age 29.90 ± 10.77 years	ADHD (*N* = 20) (10 males). non-ADHD (*N* = 20) (10 males)	Deep CNN	EEG	88%	ADHD and non-ADHD

**Notes.**

CNN convolutional neural networks EEG electroencephalogram fNIRS functional nearinfrared spectroscopy ADHD attention-deficit/hyperactivity disorder LSTM Long short-term memory fMRI functional magnetic resonance imaging RNN recurrent neural networks DBN deep belief network

CNNs have been proven to be efficient in research areas such as image recognition and classification. In recent years, many researchers have used CNNs to measure attention and try to find the neural features that correspond to it. For example, Borhani et al. (2018) developed an EEG-based classifier that used CNN to investigate underlying subject-specific features related to early visual attention. Hosseini & Guo (2019) developed a channelwise deep CNN model to classify features extracted from EEG signals from a focusing state and mind wandering. Ho et al. (2019) proposed a framework for distinguishing mental workload by combining hemoglobin concentration features with CNN. Wang, Antonenko & Dawson (2020) proposed a multiscale convolutional neural network-dynamic graph convolutional network (AMCNN-DGCN) model that estimated driving fatigue using EEG data from the driving task.

However, CNNs are not well suited to processing sequential data. Therefore, researchers developed RNN models in which neural network nodes and connections form a directed graph along a temporal sequence. As a result, RNNs are suitable for tasks involving sequential data, such as online handwriting recognition (where features can be extracted from both the pen trajectory and the resulting image) (Wu et al., 2014) and speech recognition (Fayek, Lech & Cavedon, 2017). RNNs can also be used to assess the state of sustained attention over time. Moinnereau et al. (2018) presented a deep RNN architecture for learning robust features and predicting cognitive load levels from EEG recordings. Jeong & Jeong (2020) utilized RNNs to distinguish between possible attention states. In addition, some electrophysiological data with long recording times are also suitable for processing with RNNs. For example, Phan et al. (2018) proposed a feature learning approach for single-channel automatic sleep stage classification. This approach is based on a deep bidirectional RNN with an attention mechanism. Huve, Takahashi & Hashimoto (2018) collected fNIRS signals and then used both a deep neural network (DNN) and an RNN to evaluate the impact of a driver’s mental state under various environmental conditions.

Here, we reviewed several types of computational models proposed for attention identification and evaluation. With the advancement of modern neuroimaging technology, computational models may be able to reduce labor costs while also facilitating the assessment of sustained attention. Neuroimaging data from these studies could in turn improve the accuracy of computational models and help researchers find neuromarkers that can represent the sustained attention cognitive process.

## Conclusions

This study conducted a systematic review of the research on sustained attention. There are currently only a few studies that comprehensively introduce sustained attention. Therefore, this article began by illustrating sustained attention using theoretical models, measurement methods, and neural mechanisms. Moreover, we proposed possible visual pathways of sustained attention based on the previous literatures. Subsequently, to facilitate evaluating sustained attention, this study summarized and compared various computational models related to attention classification.

Sustained attention, a fundamental component of attention, requires more in-depth research. Although the frontal cortex, cingulate cortex, and subcortex are all involved in sustained attention activities, clear pathways between these regions have not been identified. In addition, sustained attention is affected by both internal (such as mind wandering) and external factors. According to the resource-control model of sustained attention, mind wandering is unrelated to the task at hand. Although computational methods combined with neuroimaging data have high potential value, much work still needs to be done by researchers in this field. Therefore, research on sustained attention is gradually forming its characteristic framework (see Fig. S2).

There are still some limitations in the quantitative research of sustained attention. The first is that neuroimaging data are obtained by measuring the brain’s neural activity during a sustained attention task. It is worth noting, however, that the quantifications for sustained attention differ slightly across experimental paradigms. For example, the CPT3 measures inattentiveness, impulsivity, sustained attention, and vigilance (Conners, 2008), whereas the SART measures sustained attention and inhibitory control (Dinges & Powell, 1985). Because the research content of different experimental paradigms differs slightly, neural activity measured by different experimental paradigms may be biased toward different cognitive content. More research is needed in the future to verify whether the differences caused by different experimental paradigms can reflect different cognitive processing of sustained attention. Second, current computational models focus on machine learning algorithms while ignoring the neural mechanisms of sustained attention, resulting in a lack of interpretability. Therefore, in the future, more computational models for sustained attention must be designed and developed in combination with neural mechanisms (Theiss, Bowen & Silver, 2022).

Although many studies have been exploring sustained attention over the past few decades, future research is still needed to address these unclear issues. Paradigms of sustained attention measurements used in neuropsychology are not regularly used in daily life (Mueller et al., 2017). Research must develop more realistic and accurate paradigms that consider actual problems in natural settings. The neural mechanisms of sustained attention play a vital role in improving humans’ attention and task performance. Exploring the possible neural mechanisms of sustained attention can assist humans in maximizing the potential of their brains (Unsworth, Robison & Miller, 2018). In addition, analyzing the causal relationship between the neural mechanism of sustained attention and other factors, such as sleep (Veksler & Gunzelmann, 2018), working memory (Buehner et al., 2006), and environment (Kokoç, IIgaz & Altun, 2020), may provide insight into the relationship between sustained attention and cognitive task performance.

It has been previously found that many methods, such as video game training (Anguera et al., 2013), yoga courses (Ganpat, Sheela & Nagendra, 2013), mindfulness courses (Ziegler et al., 2019), neurofeedback (Bagherzadeh et al., 2020), and transcranial direct current stimulation (tDCS) (Gibson et al., 2021), combined with neural mechanisms can effectively increase humans’ sustained attention. For example, anodal tDCS of the right inferior frontal cortex resulted in an increase in attention ability (Coffman, Clark & Parasuraman, 2014). Therefore, exploring neural underpinning of sustained attention and combining them with attention therapies has a high potential to provide more effective approaches to improve sustained attention.

**Figure 3:**
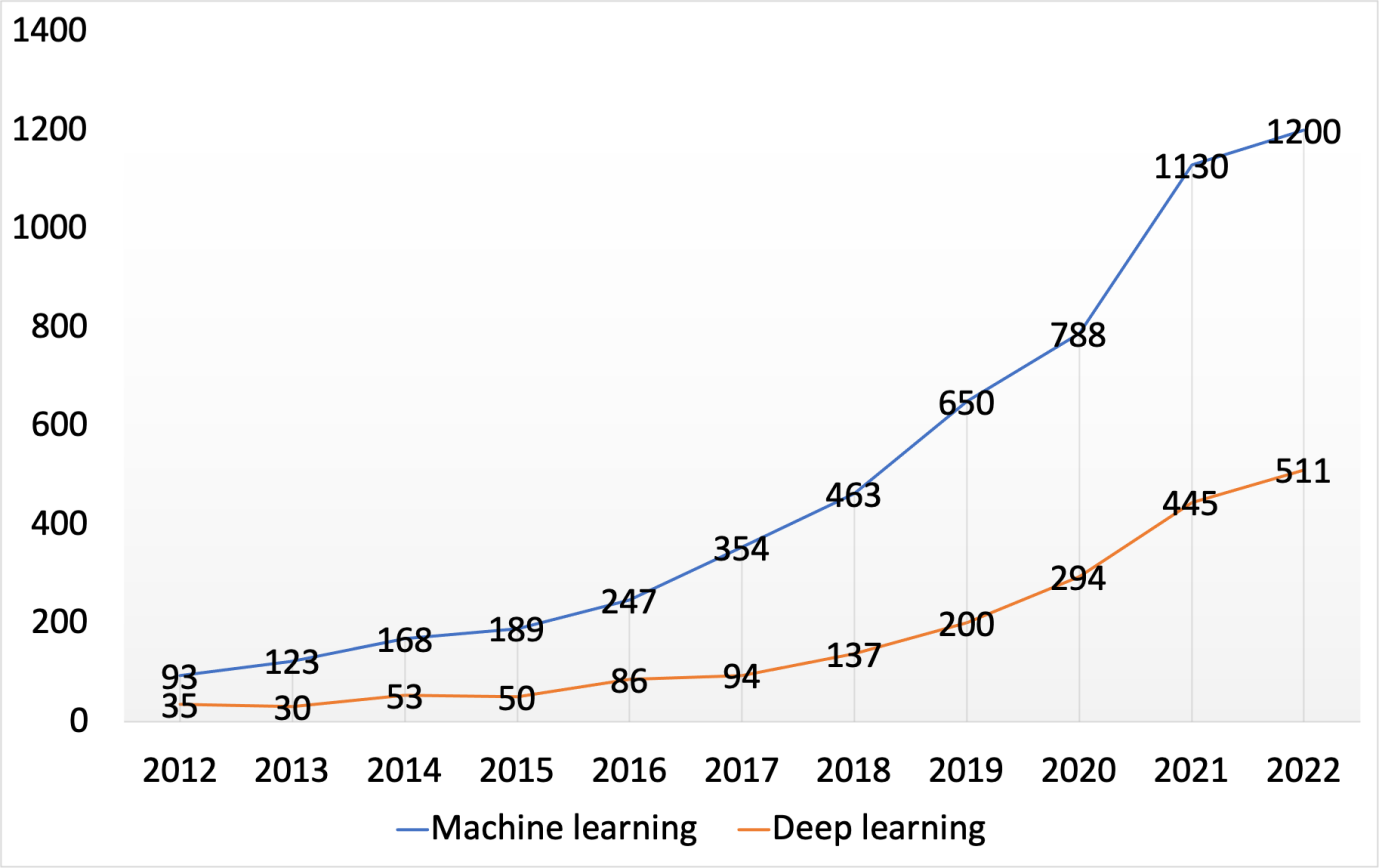
Growth of machine learning and deep learning in sustained attention. Papers involving sustained attention and computational method were identified with a search for “machine learning” or “deep learning” and ”sustained attention” on Google Scholar.

Novel machine learning methods that can process multimodal data and measure sustained attention are eagerly awaited. Machine learning is indispensable in processing data. The number of papers that used computational models to analyze and classify sustained attention increased each year from 2012 to 2022, as shown in Fig. 3. Multimodal data, such as behavioral data and neuroimaging data, can provide complementary information for measuring sustained attention (Cruz-Garza et al., 2021; Kucyi et al., 2020). Thus, it is imperative to integrate and analyze these complex data from various recording devices to accurately and robustly monitor humans’ sustained attention.

In sum, this review combines the theoretical models, neural mechanisms, and computational models on sustained attention in multiple fields to give a framework that can help researchers to understand sustained attention in as much detail as possible. Although many datasets have been obtained from the latest neuroimaging technology in the field of sustained attention, it is difficult to analyze or predict the obtained results without using computational models. In addition, rather than judging different levels of sustained attention impairment, incorporating experimental data with appropriate theoretical models can accurately interpret the obtained results from a neuroscience perspective. Therefore, this study presents many tables that analyze and compare different categories of sustained attention research for a quick overview. Considering that the visual path for sustained attention has not been fully elucidated, we propose a visual pathway based on sustained attention from related literature for the reference of researchers in this field. With the development of the world’s information technology level, future research on sustained attention will develop from cortical brain areas to deep brain areas, from single brain areas to brain networks, and from machine learning to deep learning.

##  Supplemental Information

10.7717/peerj.15351/supp-1 Supplemental Information 1 Brain regions involved in sustained attention Frontal lobe, parietal lobe, temporal lobe, and occipital lobe are divided by four colors. The circles in the figurer present the brain regions involved during sustained attention, which are mainly distributed in the frontal cortex, cingulate cortices, and subcortical structures. LPFC, lateral prefrontal cortex; pre-SMA, pre-supplementary motor area; vPMC, ventral premotor cortex; mPFC: medial prefrontal cortex; pMFC, posterior medial frontal cortex; PCC, posterior cingulate cortex.

10.7717/peerj.15351/supp-2 Supplemental Information 2 Summary of the framework of sustained attention SART, sustained attention to response task; TOVA, test of variables of attention; CPT, continuous performance test.

10.7717/peerj.15351/supp-3 Supplemental Information 3 Studies of sustained attention in frontal cortex fNIRS, functional near-infrared spectroscopy; EEG, electroencephalogram; tDCS, transcranial direct current stimulation; fMRI, functional magnetic resonance imaging; PET, positron emission tomography; SART, sustained attention to response task; mPFC, medial prefrontal cortex; ADHD, attention-deficit/hyperactivity disorder; ASD, autism spectrum disorder; DLPFC, dorsolateral prefrontal cortex; TBI, traumatic brain injury; RT, reaction times; OCD, obsessive-compulsive disorder; CPT, continuous performance test; TBI-MF, mental fatigue after mild traumatic brain injury.

10.7717/peerj.15351/supp-4 Supplemental Information 4 Studies of sustained attention in cingulate cortex PET, positron emission tomography; ACC, anterior cingulate cortex; DLPFC, dorsolateral prefrontal cortex; fMRI, functional magnetic resonance imaging; SART, sustained attention to response task; dACC, dorsal anterior cingulate; SMA, supplementary motor area; IP, inferior parietal gyrus; ADHD, attention-deficit/hyperactivity disorder; NIRS, near-infrared spectroscopy; CPT, continuous performance test; rt-fMRI, real-time fMRI; EEG, electroencephalogram; ERP, event-related potential; APOE, carriers of the Apolipoprotein E; RVIP, rapid visual information processing.

## Additional Information and Declarations

### Data Availability

The following information was supplied regarding data availability: There is no raw data because this is a literature review.

## References

[ref-1] Aggarwal S, Lamba M, Verma K, Khuttan S, Gautam H (2021). A preliminary investigation for assessing attention levels for massive online open courses learning environment using eeg signals: an experimental study. Human Behavior and Emerging Technologies.

[ref-2] Akkal D, Dum RP, Strick PL (2007). Supplementary motor area and presupplementary motor area: targets of basal ganglia and cerebellar output. Journal of Neuroscience.

[ref-3] Aly M, Turk-Browne NB (2016). Attention promotes episodic encoding by stabilizing hippocampal representations. Proceedings of the National Academy of Sciences of the United States of America.

[ref-4] Anderson JR, Bothell D, Lebiere C, Matessa M (1998). An integrated theory of list memory. Journal of Memory and Language.

[ref-5] Anguera JA, Boccanfuso J, Rintoul JL, Al-Hashimi O, Faraji F, Janowich J, Kong E, Larraburo Y, Rolle C, Johnston E, Gazzaley A (2013). Video game training enhances cognitive control in older adults. Nature.

[ref-6] Arruda JE, Amoss RT, Coburn KL, McGee H (2007). A quantitative electroencephalographic correlate of sustained attention processing. Applied Psychophysiology Biofeedback.

[ref-7] Arsintescu L, Kato KH, Cravalho PF, Feick NH, Stone LS, Flynn-Evans EE (2019). Validation of a touchscreen psychomotor vigilance task. Accident Analysis & Prevention.

[ref-8] Arsintescu L, Mulligan JB, Flynn-Evans EE (2017). Evaluation of a psychomotor vigilance task for touch screen devices. Human Factors: The Journal of the Human Factors and Ergonomics Society.

[ref-9] Aston-Jones G, Cohen JD (2005). An integrative theory of locus coeruleus-norepinephrine function: adaptive gain and optimal performance. Annual Review of Neuroscience.

[ref-10] Bagherzadeh Y, Baldauf D, Pantazis D, Desimone R (2020). Alpha synchrony and the neurofeedback control of spatial attention. Neuron.

[ref-11] Barkley RA, Knouse LE, Murphy KR (2011). Correspondence and disparity in the self-and other ratings of current and childhood ADHD symptoms and impairment in adults with ADHD. Psychological Assessment.

[ref-12] Batbat T, Güven A, Dolu N (2019). Evaluation of divided attention using different stimulation models in event-related potentials. Medical and Biological Engineering and Computing.

[ref-13] Blotenberg I, Schmidt-Atzert L (2019). Towards a process model of sustained attention tests. Journal of Intelligence.

[ref-14] Borhani S, Abiri R, Muhammad JI, Jiang Y, Zhao X (2018). EEG-based visual attentional state decoding using convolutional neural network.

[ref-15] Borji A, Sihite D, Itti L (2011). Computational modeling of top-down visual attention in interactive environments.

[ref-16] Borji A, Sihite DN, Itti L (2012a). Probabilistic learning of task-specific visual attention.

[ref-17] Borji A, Sihite DN, Itti L (2012b). Probabilistic learning of task-specific visual attention.

[ref-18] Brosowsky NP, De Gutis J, Esterman M, Smilek D, Seli P (2020). Mind wandering, motivation, and task performance over time: Evidence that motivation insulates people from the negative effects of mind wandering. Psychology of Consciousness: Theory, Research, and Practice.

[ref-19] Brunia CH (1993). Waiting in readiness: gating in attention and motor preparation. Psychophysiology.

[ref-20] Buehner M, Krumm S, Ziegler M, Pluecken T (2006). Cognitive abilities and their interplay: reasoning, crystallized intelligence, working memory components, and sustained attention. Journal of Individual Differences.

[ref-21] Buschman TJ, Kastner S (2015). From behavior to neural dynamics: an integrated theory of attention. Neuron.

[ref-22] Carroll EL, Manktelow AE, Outtrim JG, Chatfield D, Forsyth F, Hutchinson PJ, Olli T, Jussi P, Lindsay W, Barbara JS, David KM, Newcombe VFJ (2020). Influence of concomitant extracranial injury on functional and cognitive recovery from mild versus moderate to severe traumatic brain injury. Journal of Head Trauma Rehabilitation.

[ref-23] Chang SE, Lenartowicz A, Hellemann G, Uddin LQ, Bearden CE (2022). Variability in cognitive task performance in early adolescence is associated with stronger between-network anticorrelation and future attention problems. BioRxiv.

[ref-24] Chen CY, Wang CJ, Chen ELWCK, Yang YK, Wang JS, Chung PC (2010). Detecting sustained attention during cognitive work using heart rate variability.

[ref-25] Chen CM, Wu CH (2015). Effects of different video lecture types on sustained attention, emotion, cognitive load, and learning performance. Computers and Education.

[ref-26] Chen JX, Zhang PW, Mao ZJ, Huang YF, Jiang DM, Zhang YN (2019). Accurate EEG-based emotion recognition on combined features using deep convolutional neural networks. IEEE Access.

[ref-27] Chen S, Guan L, Tang JHF, Zheng Y (2021). Asymmetry in cortical and subcortical structures of the brain in children and adolescents with attention-deficit/hyperactivity disorder. Neuropsychiatric Disease and Treatment.

[ref-28] Chun MM, Golomb JD, Turk-Browne NB (2011). A taxonomy of external and internal attention. Annual Review of Psychology.

[ref-29] Cirett Galán F, Beal CR, Masthoff J, Mobasher B, Desmarais MC, Nkambou R (2012). EEG estimates of engagement and cognitive workload predict math problem solving outcomes. User modeling, adaptation, and personalization. UMAP 2012.

[ref-30] Clayton MS, Yeung N, Cohen Kadosh R (2015). The roles of cortical oscillations in sustained attention. Trends in Cognitive Sciences.

[ref-31] Coffman BA, Clark VP, Parasuraman R (2014). Battery powered thought: enhancement of attention, learning, and memory in healthy adults using transcranial direct current stimulation. Neuroimage.

[ref-32] Cohen RA (2014). The neuropsychology of attention. The neuropsychology of attention.

[ref-33] Cohen RA, Sparling-Cohen YA, O’Donnell BF (1993). The neuropsychology of attention.

[ref-34] Conners CK (2008). Conners 3.

[ref-35] Conners CK, Sitarenios G, Kreutzer JS, DeLuca J, Caplan B (2011). Conners’ Continuous Performance Test (CPT). Encyclopedia of clinical neuropsychology.

[ref-36] Connor CE, Egeth HE, Yantis S (2004). Visual attention: bottom-up versus top-down. Current Biology.

[ref-37] Conway BR (2014). Color signals through dorsal and ventral visual pathways. Visual Neuroscience.

[ref-38] Corlier J, Burnette E, Wilson AC, Lou JJ, Landeros A, Minzenberg MJ, Leuchter AF (2020). Effect of repetitive transcranial magnetic stimulation (rTMS) treatment of major depressive disorder (MDD) on cognitive control. Journal of Affective Disorders.

[ref-39] Craigmyle NA (2013). The beneficial effects of meditation: contribution of the anterior cingulate and locus coeruleus. Frontiers in Psychology.

[ref-40] Cruz-Garza JG, Darfler M, Rounds JD, Gao E, Kalantari S (2021). EEG-based investigation of the impact of classroom design on cognitive performance of students.

[ref-41] Davies DR, Parasuraman R (1982). The psychology of vigilance.

[ref-42] Dietrich A, Audiffren M (2011). The reticular-activating hypofrontality (RAH) model of acute exercise. Neuroscience and Biobehavioral Reviews.

[ref-43] Dinges DF, Kribbs NB (1991). Performing while sleepy: effects of experimentally-induced sleepiness.

[ref-44] Dinges DF, Powell JW (1985). Microcomputer analyses of performance on a portable, simple visual RT task during sustained operations. Behavior Research Methods, Instruments, Computers.

[ref-45] Dowman R, Ben-Avraham D (2008). An artificial neural network model of orienting attention toward threatening somatosensory stimuli. Psychophysiology.

[ref-46] Dubreuil-Vall L, Ruffini G, Camprodon J (2019). A deep learning approach with event-related spectral EEG data in attentional deficit hyperactivity disorder.

[ref-47] Esterman M, Grosso M, Liu G, Mitko A, Morris R, De Gutis J (2016). Anticipation of monetary reward can attenuate the vigilance decrement. PLOS ONE.

[ref-48] Esterman M, Noonan SK, Rosenberg M, Degutis J (2013). In the zone or zoning out? Tracking behavioral and neural fluctuations during sustained attention. Cerebral Cortex.

[ref-49] Esterman M, Poole V, Liu G, De Gutis J (2017). Modulating reward induces differential neurocognitive approaches to sustained attention. Cerebral Cortex.

[ref-50] Esterman M, Rothlein D (2019). Models of sustained attention. Current Opinion in Psychology.

[ref-51] Fan J, Gan J, Liu W, Zhong M, Liao H, Zhang HYJ, Chan RCK, Tan C, Zhu X (2018). Resting-state default mode network related functional connectivity is associated with sustained attention deficits in schizophrenia and obsessive-compulsive disorder. Frontiers in Behavioral Neuroscience.

[ref-52] Fan J, Hof PR, Guise KG, Fossella JA, Posner MI (2008). The functional integration of the anterior cingulate cortex during conflict processing. Cerebral Cortex.

[ref-53] Fan J, McCandliss BD, Fossella J, Flombaum JI, Posner MI (2005). The activation of attentional networks. NeuroImage.

[ref-54] Fayek HM, Lech M, Cavedon L (2017). Evaluating deep learning architectures for Speech Emotion Recognition. Neural Networks.

[ref-55] Fortenbaugh FC, De Gutis J, Esterman M (2017). Recent theoretical, neural, and clinical advances in sustained attention research. Annals of the New York Academy of Sciences.

[ref-56] Fortenbaugh FC, Rothlein D, McGlinchey R, De Gutis J, Esterman M (2018). Tracking behavioral and neural fluctuations during sustained attention: a robust replication and extension. NeuroImage.

[ref-57] Fouladvand S, Hankosky ER, Henderson DW, Bush H, Chen J, Dwoskin LP, Freeman PR, Kantak K, Talbert J, Tao S, Zhang GQ (2018). June Predicting substance use disorder in ADHD patients using long-short term memory model.

[ref-58] Francisco-Vicencio MA, Góngora-Rivera F, Ortiz-Jiménez X, Martinez-Peon D (2022). Sustained attention variation monitoring through EEG effective connectivity. Biomedical Signal Processing and Control.

[ref-59] Fu Z, Beam D, Chung JM, Reed CM, Mamelak AN, Adolphs R, Rutishauser U (2022). The geometry of domain-general performance monitoring in the human medial frontal cortex. Science.

[ref-60] Ganpat T, Sheela, Nagendra HR (2013). Efficacy of Yoga for sustained attention in university students. AYU (An International Quarterly Journal of Research in Ayurveda).

[ref-61] Gartenberg D, Gunzelmann G, Hassanzadeh-Behbaha S, Trafton JG (2018). Examining the role of task requirements in the magnitude of the vigilance decrement. Frontiers in Psychology.

[ref-62] Ghassemi F, Hassan_Moradi M, Tehrani-Doost M, Abootalebi V (2012). Using non-linear features of EEG for ADHD/normal participants’ classification. Procedia-Social and Behavioral Sciences.

[ref-63] Ghassemi F, Moradi MH, Doust MT, Abootalebi V (2009). Combination of independent component analysis and feature extraction of ERP for level classification of sustained attention.

[ref-64] Ghassemi F, Moradi MH, Tehrani-Doost M, Abootalebi V (2010). Effects of correct and wrong answers on ERPs recorded under conditions of the continuous performance test in ADHD/normal participants. Neurophysiology.

[ref-65] Gibson BC, Heinrich M, Mullins TSYAB, Hansberger JT, Clark VP (2021). Baseline differences in anxiety affect attention and tDCS-mediated learning. Frontiers in Human Neuroscience.

[ref-66] Greenlee ET, Hess LJ (2019). Stress linked to reports of simulator sickness in vigilance: a factor analysis of the SSSQ and the SSQ. Proceedings of the Human Factors and Ergonomics Society Annual Meeting.

[ref-67] Gunzelmann G, Richard Moore L, Salvucci DD, Gluck KA (2011). Sleep loss and driver performance: quantitative predictions with zero free parameters. Cognitive Systems Research.

[ref-68] Gurudath N, Bryan Riley H (2014). Drowsy driving detection by EEG analysis using Wavelet Transform and K-means clustering. Procedia Computer Science.

[ref-69] Han HB, Lee KE, Choi JH (2019). Functional dissociation of *θ* oscillations in the frontal and visual cortices and their long-range network during sustained attention. ENeuro.

[ref-70] Hancock PA (1989). A dynamic model of stress and sustained attention. Human Factors.

[ref-71] Hancock PA, Warm JS (2003). A dynamic model of stress and sustained attention. Journal of Human Performance in Extreme Environments.

[ref-72] Hao AJHBL, Yin CH (2015). Discrimination of ADHD children based on Deep Bayesian Network.

[ref-73] Helfrich RF, Fiebelkorn IC, Szczepanski SM, Lin JJ, Parvizi J, Knight RT, Kastner S (2018). Neural mechanisms of sustained attention are rhythmic. Neuron.

[ref-74] Helton WS, Russell PN (2012). Brief mental breaks and content-free cues may not keep you focused. Experimental Brain Research.

[ref-75] Ho TKK, Gwak J, Park CM, Khare A, Song JI (2019). Deep leaning-based approach for mental workload discrimination from multi-channel fNIRS. Lecture Notes in Electrical Engineering.

[ref-76] Hommel B, Chapman CS, Cisek P, Neyedli HF, Song JH, Welsh TN (2019). No one knows what attention is. Attention, Perception, and Psychophysics.

[ref-77] Hosseini S, Guo X (2019). Deep convolutional neural network for automated detection of mind wandering using EEG signals.

[ref-78] Hu BLX, Sun S, Ratcliffe M (2016). Attention recognition in EEG-based affective learning research using CFS+ KNN algorithm. IEEE/ACM Transactions on Computational Biology and Bioinformatics.

[ref-79] Hursh SR, Redmond DP, Johnson ML, Thorne DR, Belenky G, Balkin TJ, Storm WF, Miller JCED (2004). Fatigue models for applied research in warfighting. Aviation, Space, and Environmental Medicine.

[ref-80] Huve G, Takahashi K, Hashimoto M, Køurková V, Manolopoulos Y, Hammer B, Iliadis L, Maglogiannis I (2018). fNIRS-based brain–computer interface using deep neural networks for classifying the mental state of drivers. Artificial neural networks and machine learning—ICANN 2018. ICANN 2018.

[ref-81] Itti L, Baldi P (2005). Bayesian surprise attracts human attention. Advances in neural information processing systems 18 (NIPS 2005).

[ref-82] Jackson ML, Gunzelmann G, Whitney P, Hinson JM, Belenky G, Rabat A, Van Dongen HPA (2013). Deconstructing and reconstructing cognitive performance in sleep deprivation. Sleep Medicine Reviews.

[ref-83] Jafari Z, Malayeri S, Rostami R (2015). Subcortical encoding of speech cues in children with attention deficit hyperactivity disorder. Clinical Neurophysiology.

[ref-84] Jagtap P, Diwadkar VA (2016). Effective connectivity of ascending and descending frontalthalamic pathways during sustained attention: Complex brain network interactions in adolescence. Human Brain Mapping.

[ref-85] Jeong DH, Jeong J (2020). In-ear EEG based attention state classification using echo state network. Brain Sciences.

[ref-86] Jewett ME, Kronauer RE (1999). Interactive mathematical models of subjective alertness and cognitive throughput in humans. Journal of Biological Rhythms.

[ref-87] Jin CY, Borst JP, van Vugt MK (2020). Distinguishing vigilance decrement and low task demands from mind-wandering: a machine learning analysis of EEG. European Journal of Neuroscience.

[ref-88] Jo T, Nho K, Saykin AJ (2019). Deep learning in Alzheimer’s disease: diagnostic classification and prognostic prediction using neuroimaging data. Frontiers in Aging Neuroscience.

[ref-89] Jones AD, Cho RY, Nystrom LE, Cohen JD, Braver TS (2002). A computational model of anterior cingulate function in speeded response tasks: effects of frequency, sequence, and conflict. Cognitive, Affective and Behavioral Neuroscience.

[ref-90] Kaewkhaw R, Kaya KD, Brooks M, Homma K, Zou J, Chaitankar V, Rao M, Swaroop A (2015). Transcriptome dynamics of developing photoreceptors in three-dimensional retina cultures recapitulates temporal sequence of human cone and rod differentiation revealing cell surface markers and gene networks. Stem Cells.

[ref-91] Kass SJ, Wallace JC, Vodanovich SJ (2003). Boredom proneness and sleep disorders as predictors of adult attention deficit scores. Journal of Attention Disorders.

[ref-92] Khullar V, Salgotra K, Singh HP, Sharma DP (2021). Deep learning-based binary classification of ADHD using resting state MR images. Augmented Human Research.

[ref-93] Klimesch W (2012). Alpha-band oscillations, attention, and controlled access to stored information. Trends in Cognitive Sciences.

[ref-94] Kokoç M, IIgaz H, Altun A (2020). Effects of sustained attention and video lecture types on learning performances. Educational Technology Research and Development.

[ref-95] Kral TRA, Imhoff-Smith T, Dean DC, Grupe D, Adluru N, Patsenko E, Mumford JA, Goldman R, Rosenkranz MA, Davidson RJ (2019). Mindfulness-based stress reduction-related changes in posterior cingulate resting brain connectivity. Social Cognitive and Affective Neuroscience.

[ref-96] Kronauer RE, Forger DB, Jewett ME (1999). Quantifying human circadian pacemaker response to brief, extended, and repeated light stimuli over the phototopic range. Journal of Biological Rhythms.

[ref-97] Kropp P, Linstedt U, Niederberger U, Gerber W-D (2001). Contingent negative variation and attentional performance in humans. Neurological Research.

[ref-98] Kuang D, Guo XAX, Zhao Y, He L, Huang DS, Han K, Gromiha M (2014). Discrimination of ADHD based on fMRI data with deep belief network. Intelligent computing in bioinformatics. ICIC 2014.

[ref-99] Kuang D, He L (2014). Classification on ADHD with deep learning.

[ref-100] Kucyi A, Daitch A, Raccah O, Zhao B, Zhang C, Esterman M, Zeineh M, Halpern CH, Zhang K, Zhang J, Parvizi J (2020). Electrophysiological dynamics of antagonistic brain networks reflect attentional fluctuations. Nature Communications.

[ref-101] Kurzban R, Duckworth A, Kable JW, Myers J (2013). An opportunity cost model of subjective effort and task performance. Behavioral and Brain Sciences.

[ref-102] Langner R, Eickhoff SB (2013). Sustaining attention to simple tasks: a meta-analytic review of the neural mechanisms of vigilant attention. Psychological Bulletin.

[ref-103] Larue GS, Rakotonirainy A, Pettitt AN (2010). Real-time performance modelling of a sustained attention to response task. Ergonomics.

[ref-104] Lau WKW, Leung MK, Zhang R (2020). Hypofunctional connectivity between the posterior cingulate cortex and ventromedial prefrontal cortex in autism: evidence from coordinate-based imaging meta-analysis. Progress in Neuro-Psychopharmacology and Biological Psychiatry.

[ref-105] Lauritzen TZ, D’Esposito M, Heeger DJ, Silver MA (2009). Top–down flow of visual spatial attention signals from parietal to occipital cortex. Journal of Vision.

[ref-106] Leech R, Sharp DJ (2014). The role of the posterior cingulate cortex in cognition and disease. Brain.

[ref-107] Lenartowicz A, Simpson GV, Cohen MS (2013). Perspective: causes and functional significance of temporal variations in attention control. Frontiers in Human Neuroscience.

[ref-108] Li J, Kronemer SI, Herman WX, Kwon H, Ryu JH, Micek CWY, Gerrard J, Spencer DD, Blumenfeld H (2019). Default mode and visual network activity in an attention task: direct measurement with intracranial EEG. Neuroimage.

[ref-109] Lim J, Wu W chau, Wang J, Detre JA, Dinges DF, Rao H (2010). Imaging brain fatigue from sustained mental workload: an ASL perfusion study of the time-on-task effect. NeuroImage.

[ref-110] Lin HY, Chang WD, Hsieh HCYWH, Lee P (2021). Relationship between intraindividual auditory and visual attention in children with ADHD. Research in Developmental Disabilities.

[ref-111] Liu JSY, Liu Y (2017). Multi-modal emotion recognition with temporal-band attention based on LSTM-RNN. In Pacific rim conference on multimedia.

[ref-112] Liu JYTWJ, Pan Y, Tan Z, Liu R, Wang X, Ren L, Wang L (2021). Anterior thalamic stimulation improves working memory precision judgments. Brain Stimulation.

[ref-113] Loetscher T, Potter K, Wong D, Nair Rdas (2019). Cognitive rehabilitation for attention deficits following stroke. Cochrane Database of Systematic Reviews.

[ref-114] Lohr I (1999). The influence of effort on impairments of attention associated with major affective disorders. MA Thesis in Clinical Psychology.

[ref-115] Loitfelder M, Filippi M, Rocca M, Valsasina P, Ropele S, Jehna M, Fuchs S, Schmidt R, Neuper C, Fazekas F, Enzinger C (2012). Abnormalities of resting state functional connectivity are related to sustained attention deficits in MS. PLOS ONE.

[ref-116] Long NM, Kuhl BA (2018). Bottom-up and top-down factors differentially influence stimulus representations across large-scale attentional networks. Journal of Neuroscience.

[ref-117] Luo Y, X.feng Chen, Zhang Y, Chen X, XY Liu, TK Fan (2020). Visual focus of attention estimation based on improved hybrid incremental dynamic Bayesian network. Optoelectronics Letters.

[ref-118] Luria AR (1973). The frontal lobes and the regulation of behavior. Psychophysiology of the Frontal Lobes.

[ref-119] Machner B, Braun L, Imholz J, Koch PJ, Münte TF, Helmchen C, Sprenger A (2022). Resting-state functional connectivity in the dorsal attention network relates to behavioral performance in spatial attention tasks and may show task-related adaptation. Frontiers in Human Neuroscience.

[ref-120] Mackworth NH (1948). The breakdown of vigilance during prolonged visual search. Quarterly Journal of Experimental Psychology.

[ref-121] Mackworth NH (1950). Researches on the measurement of human performance (Medical Research Council Special Report Series No. 268).

[ref-122] Malkovsky E, Merrifield C, Goldberg Y, Danckert J (2012). Exploring the relationship between boredom and sustained attention. Experimental Brain Research.

[ref-123] Manly T, Robertson IH (2005). The sustained attention to response test (SART). Neurobiology of attention.

[ref-124] Mansour R, Ward AR, Lane DM, Loveland KA, Aman MG, Jerger S, Schachar RJ, Pearson DA (2021). ADHD severity as a predictor of cognitive task performance in children with autism spectrum disorder (ASD). Research in Developmental Disabilities.

[ref-125] Mao Z, Su Y, Xu G, Wang X, Huang Y, Yue W, Sun L, Xiong N (2019). Spatio-temporal deep learning method for adhd fmri classification. Information Sciences.

[ref-126] McAlonan K, Cavanaugh J, Wurtz RH (2008). Guarding the gateway to cortex with attention in visual thalamus. Nature.

[ref-127] Miao B, Zhang Y (2017). A feature selection method for classification of ADHD.

[ref-128] Ming Y, Wang YK, Prasad MWD, Lin CT (2018). Sustained attention driving task analysis based on recurrent residual neural network using EEG data.

[ref-129] Mitko A, Rothlein D, Poole V, Robinson M, McGlinchey R, De Gutis J, Salat D, Esterman M (2019). Individual differences in sustained attention are associated with cortical thickness. Human Brain Mapping.

[ref-130] Moinnereau MA, Brienne T, Brodeur S, Rouat J, Whittingstall K, Plourde E (2018). Classification of auditory stimuli from EEG signals with a regulated recurrent neural network reservoir.

[ref-131] Mueller A, Hong DS, Shepard S, Moore T (2017). Linking ADHD to the neural circuitry of attention. Trends in Cognitive Sciences.

[ref-132] Munir F, Cornish KM, Wilding J (2000). A neuropsychological profile of attention deficits in young males with fragile X syndrome. Neuropsychologia.

[ref-133] Munnik A, Näswall K, Woodward G, Helton WS (2020). The quick and the dead: a paradigm for studying friendly fire. Applied Ergonomics.

[ref-134] Nelson AJD (2021). The anterior thalamic nuclei and cognition: a role beyond space?. Neuroscience & Biobehavioral Reviews.

[ref-135] Offen S, Gardner JL, Schluppeck D, Heeger DJ (2010). Differential roles for frontal eye fields (FEFs) and intraparietal sulcus (IPS) in visual working memory and visual attention. Journal of Vision.

[ref-136] Okabe H (2016). Exploring the role of the dorsal attention network in sustained attention with rTMS.

[ref-137] Oliveira CR de, Pedron AC, Gurgel LG, Reppold CT, Fonseca RP (2012). Executive functions and sustained attention: comparison between age groups of 19–39 and 40–59 years old. Dementia & Neuropsychologia.

[ref-138] Öztoprak H, Toycan M, Alp YK, Arıkan O, Doğutepe E, Karakaş S (2017). Machine-based classification of ADHD and nonADHD participants using time/frequency features of event-related neuroelectric activity. Clinical Neurophysiology.

[ref-139] Pamplona GSP, Heldner J, Langner R, Koush Y, Michels L, Ionta S, Scharnowski F, Salmon CEG (2020). Network-based fMRI-neurofeedback training of sustained attention. Neuroimage.

[ref-140] Pang D, Kimura A, Takeuchi T, Yamato J, Kashino K (2008). A stochastic model of selective visual attention with a dynamic Bayesian network.

[ref-141] Parasuraman R, Mutter SA, Molloy R (1991). Sustained attention following mild closed-head injury. Journal of Clinical and Experimental Neuropsychology.

[ref-142] Phan H, Andreotti F, Cooray N, Chen OY, Vos M De (2018). Automatic sleep stage classification using single-channel EEG: learning sequential features with attention-based recurrent neural networks.

[ref-143] Pinto Y, van der Leij AR, Sligte IG, Lamme VAF, Scholte HS (2013). Bottom-up and top-down attention are independent. Journal of Vision.

[ref-144] Piper RJ, Richardson RM, Worrell G, Carmichael DW, Baldeweg T, Litt B, Denison T, Tisdall MM (2022). Towards network-guided neuromodulation for epilepsy. Brain.

[ref-145] Rajab AS, Crane DE, Middleton LE, Robertson AD, Hampson M, MacIntosh BJ (2014). A single session of exercise increases connectivity in sensorimotor-related brain networks: a resting-state fMRI study in young healthy adults. Frontiers in Human Neuroscience.

[ref-146] Rapoport JL, Buchsbaum MS, Zahn TP, Weingartner H, Ludlow C (1978). Dextroamphetamine: cognitive and behavioral effects. Science.

[ref-147] Rasyid MFA, Djamal EC (2019). Emotion and attention of neuromarketing using wavelet and recurrent neural networks. International conference on electrical engineering, computer science and informatics (EECSI).

[ref-148] Raver CC, Blair C (2016). Neuroscientific insights: attention, working memory, and inhibitory control. The Future of Children.

[ref-149] Robertson IH, Manly T, Andrade J, Baddeley BT, Yiend J (1997). ’Oops!’: performance correlates of everyday attentional failures in traumatic brain injured and normal subjects. Neuropsychologia.

[ref-150] Rubia K, Criaud M, Wulff M, Alegria A, Brinson H, Barker G, Stahl D, Giampietro V (2019). Functional connectivity changes associated with fMRI neurofeedback of right inferior frontal cortex in adolescents with ADHD. NeuroImage.

[ref-151] Samaha J, Sprague TC, Postle BR (2016). Decoding and reconstructing the focus of spatial attention from the topography of alpha-band oscillations. Journal of Cognitive Neuroscience.

[ref-152] Sarter M, Givens B, Bruno JP (2001). The cognitive neuroscience of sustained attention: where top-down meets bottom-up. Brain Research Reviews.

[ref-153] Saygin AP, Sereno MI (2008). Retinotopy and attention in human occipital, temporal, parietal, and frontal cortex. Cerebral Cortex.

[ref-154] Scheinost D, Hsu TW, Avery EW, Hampson M, Constable RT, Chun MM, Rosenberg MD (2020). Connectome-based neurofeedback: a pilot study to improve sustained attention. NeuroImage.

[ref-155] See JE, Howe SR, Warm JS, Dember WN (1995). Meta-analysis of the sensitivity decrement in vigilance. Psychological Bulletin.

[ref-156] Segalowitz SJ, Dywan J, Unsal A (1997). Attentional factors in response time variability after traumatic brain injury: an ERP study. Journal of the International Neuropsychological Society.

[ref-157] Shoeibi A, Ghassemi N, Khodatars M, Moridian P, Khosravi A, Zare A, Gorriz JM, Chale-Chale AH, Khadem A, Acharya UR (2022). Automatic diagnosis of schizophrenia and attention deficit hyperactivity disorder in rs-fMRI modality using convolutional autoencoder model and interval type-2 fuzzy regression. Cognitive Neurodynamics.

[ref-158] Shumskaya E, Andriessen TMJC, Norris DG, Vos PE (2012). Abnormal whole-brain functional networks in homogeneous acute mild traumatic brain injury. Neurology.

[ref-159] Silver MA, Ress D, Heeger DJ (2007). Neural correlates of sustained spatial attention in human early visual cortex. Journal of Neurophysiology.

[ref-160] Sonuga-Barke EJS, Castellanos FX (2007). Spontaneous attentional fluctuations in impaired states and pathological conditions: a neurobiological hypothesis. Neuroscience and Biobehavioral Reviews.

[ref-161] Terashima H, Kihara K, Kawahara JI, Kondo HM (2021). Common principles underlie the fluctuation of auditory and visual sustained attention. Quarterly Journal of Experimental Psychology.

[ref-162] Theiss JD, Bowen JD, Silver MA (2022). Spatial attention enhances crowded stimulus encoding across modeled receptive fields by increasing redundancy of feature representations. Neural Computation.

[ref-163] Thillay A, Roux S, Gissot V, Carteau-Martin I, Knight RT, Bonnet-Brilhault F, Bidet-Caulet A (2015). Sustained attention and prediction: distinct brain maturation trajectories during adolescence. Frontiers in Human Neuroscience.

[ref-164] Thompson C, Fransen J, Beavan A, Skorski S, Coutts A, Meyer T (2020b). Understanding the influence of a cognitively demanding task on motor response times and subjective mental fatigue/boredom. Brazilian Journal of Motor Behavior.

[ref-165] Thompson CJ, Noon M, Towlson C, Perry J, Coutts AJ, Harper LD, Skorski SS, Smith MR, Barrett S, Meyer T (2020a). Understanding the presence of mental fatigue in English academy soccer players. Journal of Sports Sciences.

[ref-166] Thomson DR, Besner D, Smilek D (2015). A resource-control account of sustained attention: evidence from mind-wandering and vigilance paradigms. Perspectives on Psychological Science.

[ref-167] Tokoro K, Sato H, Yamamoto M, Nagai Y (2015). Thalamus and attention. Brain and Nerve.

[ref-168] Torres VA, Ashford JM, Wright EXJ, Zhang H, Merchant TE, Conklin HM (2021). The impact of socioeconomic status (SES) on cognitive outcomes following radiotherapy for pediatric brain tumors: a prospective, longitudinal trial. Neuro-Oncology.

[ref-169] Treichler DG (1967). Are you missing the boat in training aids? Film and Audio-Visual Communication (First edition).

[ref-170] Tu M-C, Hsu Y-H, Yang J-J, Huang W-H, Deng JF Lin, S-Y, Lin C-Y, Kuo L-W (2020). Attention and functional connectivity among patients with early-stage subcortical ischemic vascular disease and Alzheimer’s disease. Frontiers in Aging Neuroscience.

[ref-171] Unsworth N, Robison MK, Miller AL (2018). Pupillary correlates of fluctuations in sustained attention. Journal of Cognitive Neuroscience.

[ref-172] Vahid A, Bluschke A, Roessner V, Stober S, Beste C (2019). Deep learning based on event-related EEG differentiates children with adhd from healthy controls. Journal of Clinical Medicine.

[ref-173] van ESMWJ, Marshall TR, Spaak E, Jensen O, Schoffelen J (2022). Phasic modulation of visual representations during sustained attention. European Journal of Neuroscience.

[ref-174] Vaughn-Coaxum RA, Merranko J, Birmaher B, Goldstein TR, Dickstein DP, Hafeman D, Levenson JC, Liao F, Gill MK, Hower H, Goldstein BI, Strober M, Ryan ND, Diler R, Keller MB, Yen S, Weinstock LM, Axelson D, Goldstein TR (2021). Longitudinal course of depressive symptom severity among youths with bipolar disorders: moderating influences of sustained attention and history of child maltreatment. Journal of Affective Disorders.

[ref-175] Veksler BZ, Gunzelmann G (2018). Functional equivalence of sleep loss and time on task effects in sustained attention. Cognitive Science.

[ref-176] Walda S, van Weerdenburg M, vander Ven A, Bosman A (2021). Literacy progress in children with dyslexia and the role of attention. Reading and Writing Quarterly.

[ref-177] Walsh G (2014). Biopharmaceutical benchmarks 2014. Nature Biotechnology.

[ref-178] Wang J, Antonenko P, Dawson K (2020). Does visual attention to the instructor in online video affect learning and learner perceptions? An eye-tracking analysis. Computers and Education.

[ref-179] Wang Z, Myers KG, Guo Y, Ocampo MA, Pang RD, Jakowec MW, Holschneider DP (2013b). Functional reorganization of motor and limbic circuits after exercise training in a rat model of bilateral parkinsonism. PLOS ONE.

[ref-180] Warm JS (1984). Sustained attention in human performance.

[ref-181] Warm JS, Dember WN, Hancock PA, Parasuraman R, Mouloua M (1996). Vigilance and workload in automated systems. Automation and human performance: theory and applications.

[ref-182] Weigard A, Huang-Pollock C, Brown S (2016). Evaluating the consequences of impaired monitoring of learned behavior in attention-deficit/hyperactivity disorder using a Bayesian hierarchical model of choice response time. Neuropsychology.

[ref-183] Wiesendanger R, Wiesendanger M (1985). The thalamic connections with medial area 6 (supplementary motor cortex) in the monkey (macaca fascicularis). Experimental Brain Research.

[ref-184] Wilkins AJ, Shallice T, McCarthy R (1987). Frontal lesions and sustained attention. Neuropsychologia.

[ref-185] Williams LM (2016). Precision psychiatry: a neural circuit taxonomy for depression and anxiety. The Lancet Psychiatry.

[ref-186] Wong SM, Arski ON, Warsi NM, Pang EW, Kerr E, Smith ML, Dunkley BT, Ochi A, Otsubo H, Sharma R, Jain P, Donner E, Snead OC, Ibrahim GM (2022). Phase resetting in the anterior cingulate cortex subserves childhood attention and is impaired by epilepsy. Cerebral Cortex.

[ref-187] Wu C, Fan WHY, Sun J, Naoi S (2014). Handwritten character recognition by alternately trained relaxation convolutional neural network.

[ref-188] Yeo MVMLX, Shen K, Wilder-Smith EPV (2009). Can SVM be used for automatic EEG detection of drowsiness during car driving?. Safety Science.

[ref-189] Zaharchuk G, Gong E, Wintermark M, Rubin D, Langlotz CP (2018). Deep learning in neuroradiology. American Journal of Neuroradiology.

[ref-190] Zeller R (2022). Driver fatigue and performance decrements over time-on-task: effects and mitigation. PhD thesis.

[ref-191] Zhang W, Zhang Y, Zhang Q, Xu J (2022). Sustained attention states recognition with EEG and eye-tracking in the GradCPT.

[ref-192] Zhang Z, Jiao X, Jiang J, Pan J, Cao Y, Yang H, Xu F, Liu CL, Hussain A, Luo B, Tan K, Zeng Y, Zhang Z (2016). Passive BCI based on sustained attention detection: an fNIRS study. Advances in brain inspired cognitive systems. BICS 2016.

[ref-193] Zhou HX, Chen X, Shen YQLL, Chen NX, Zhu ZC, Castellanos FX, Yan CG (2020). Rumination and the default mode network: meta-analysis of brain imaging studies and implications for depression. NeuroImage.

[ref-194] Ziegler DA, Simon AJ, Gallen CL, Skinner S, Janowich JR, Volponi JJ, Rolle CE, Mishra J, Kornfield J, Anguera JA, Gazzaley A (2019). Closed-loop digital meditation improves sustained attention in young adults. Nature Human Behaviour.

[ref-195] Zou L, Zheng J, Miao C, Mckeown MJ, Wang ZJ (2017). 3D CNN based automatic diagnosis of attention deficit hyperactivity disorder using functional and structural MRI. IEEE Access.

