# Supplementary figures and images for: A review of visual sustained attention: neural mechanisms and computational models
Source: PeerJ. 2023 Jun 13;11:e15351. doi: 10.7717/peerj.15351 (PMC10274610; doi:10.7717/peerj.15351)

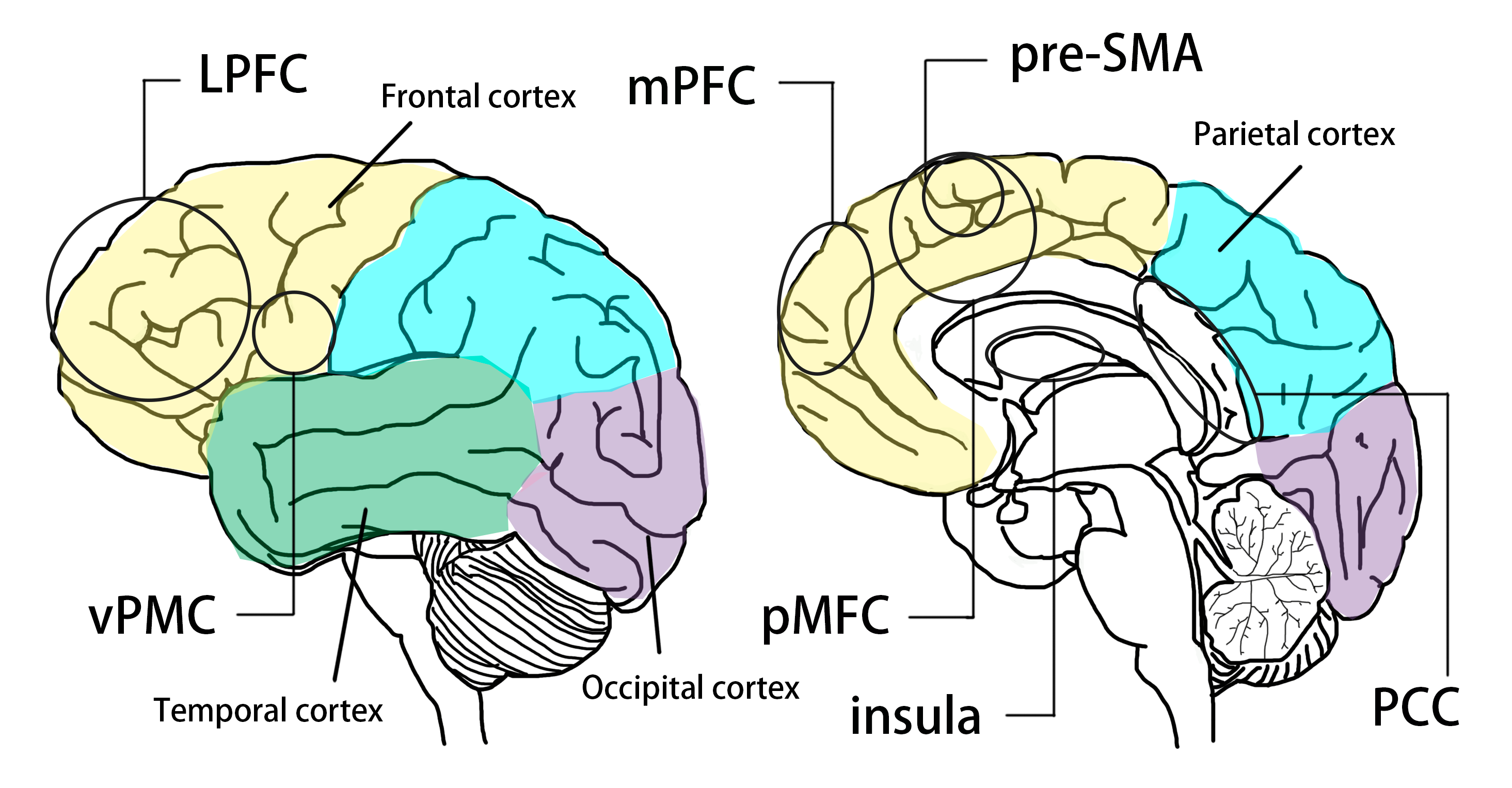

Supplement: Supplemental Information 1 — Frontal lobe, parietal lobe, temporal lobe, and occipital lobe are divided by four colors. The circles in the figurer present the brain regions involved during sustained attention, which are mainly distributed in the frontal cortex, cingulate cortices, and subcortical structures. LPFC, lateral prefrontal cortex; pre-SMA, pre-supplementary motor area; vPMC, ventral premotor cortex; mPFC: medial prefrontal cortex; pMFC, posterior medial frontal cortex; PCC, posterior cingulate cortex. [file peerj-11-15351-s001.png]

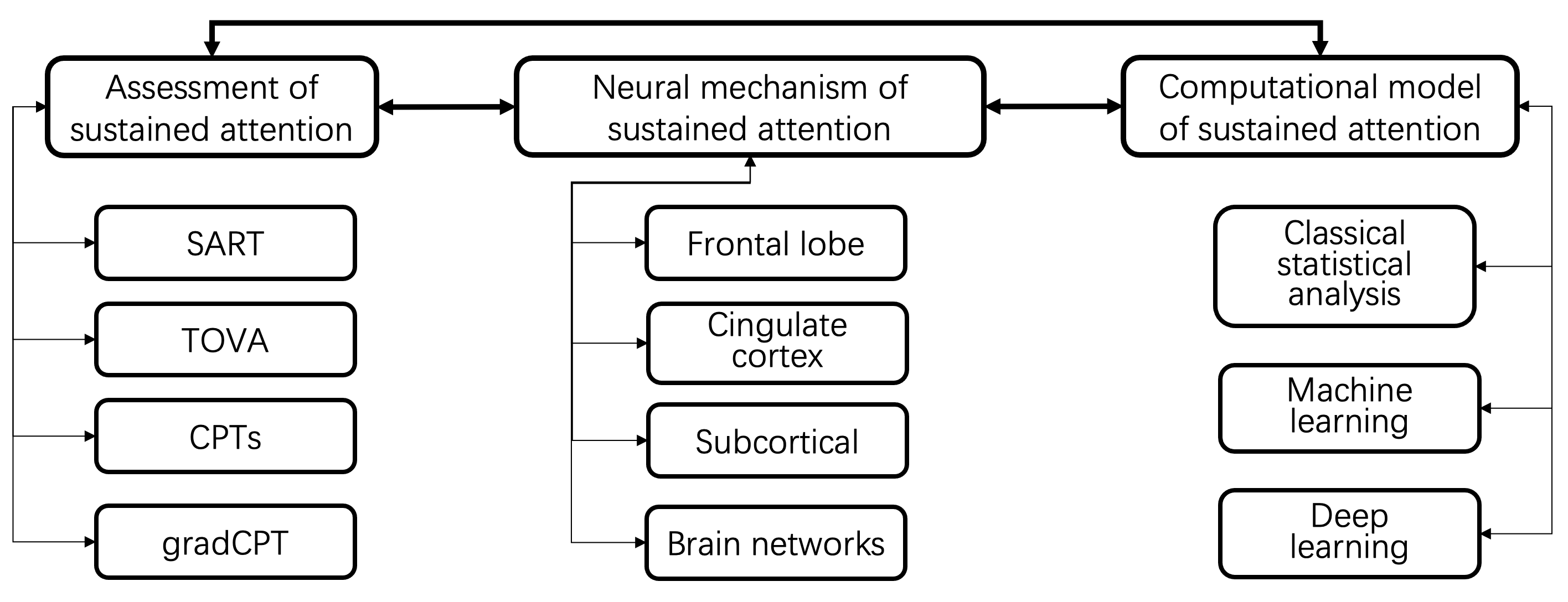

Supplement: Supplemental Information 2 — SART, sustained attention to response task; TOVA, test of variables of attention; CPT, continuous performance test. [file peerj-11-15351-s002.png]
