# Supplementary material for: A review of visual sustained attention: neural mechanisms and computational models
Source: PeerJ. 2023 Jun 13;11:e15351. doi: 10.7717/peerj.15351 (PMC10274610; doi:10.7717/peerj.15351)
Supplement: Supplemental Information 3 — fNIRS, functional near-infrared spectroscopy; EEG, electroencephalogram; tDCS, transcranial direct current stimulation; fMRI, functional magnetic resonance imaging; PET, positron emission tomography; SART, sustained attention to response task; mPFC, medial prefrontal cortex; ADHD, attention-deficit/hyperactivity disorder; ASD, autism spectrum disorder; DLPFC, dorsolateral prefrontal cortex; TBI, traumatic brain injury; RT, reaction times; OCD, obsessive-compulsive disorder; CPT, continuous performance test; TBI-MF, mental fatigue after mild traumatic brain injury. [file peerj-11-15351-s003.docx]

| **Author** | **Type of subjects** | **Number of subjects** | **Research method** | **Neuropsychological tests** | **Main ﬁndings** |
| --- | --- | --- | --- | --- | --- |
| De Joux et al. (2017) | Healthy, age 23 ±2.3 years | N=45 (21 males) | fNIRS | SART | Activation in the right pre-frontal cortex compared to the left prefrontal cortex during the task. |
| Van Schouwenburg et al. (2019) | Healthy, age 22.3±2.7 years | N=97 (26 males) | tDCS | Vigilant attention task | Enhance vigilant attention by applying transcranial electrical current stimulation over the mPFC. |
| Robertson et al. (1997) | TBI, age 34±11 years | N=75 (12 males) | Behavioral | SART | High correlations between frontal lobe and damage and relative-reported everyday attention failures. |
| Christakou et al. (2013) | ADHD, ASD, Control, age 11-17 years | ADHD (N=20), ASD (N=20), Control (N=20), 60 males | fMRI | Sustained attention task | ADHD and ASD boys had signiﬁcantly reduced activation relative to controls in bilateral striato–thalamic regions, left DLPFC and superior parietal cortex. |
| Shallice et al. (2008) | Patients with frontal cortex lesion, age 46±3 years | N=43 | Behavioral | Sustained counting | Lesions of right lateral frontal cortex impair on-going modulation of behavior by reducing monitoring capacity which we previously proposed to be controlled by systems in the RL region. |
| Mathias et al. (2004) | TBI, Control, 32.4±12.1 years | Control, (N=40) (32 males), TBI (N=40) (32 males) | Behavioral | RT task | The mild TBI group demonstrated deﬁcits in attention, non-verbal ﬂuency, and verbal memory. They also demonstrated slower visual and tactile RT. |
| Manly et al. (2003) | TBI, Control, 28.74±10.53 years | TBI (N=19) (16 males), Control, (N=16) (13 males) | PET | SART | The right prefrontal region can be affected by TBI resulting in more transitory lapses of attention during dull and ostensibly unchallenging activities. |
| Norman et al. (2017) | ADHD, OCD, Control, age 12-18 years | ADHD (N=20), OCD (N=20), Control (N=20), 60 males | fMRI | Sustained attention task | ADHD and OCD patients showed mostly disorder-speciﬁc patterns of brain abnormalities in both task positive salience/ventral attention networks with lateral frontal deﬁcits in ADHD. |
| Negoro et al. (2010) | ADHD, Control, age 6-13 years | ADHD (N=20) (18 males), Control (N=20) (17 males) | NIRS | Stroop color-word task | During the task, the oxygen–hemoglobin changes in the control group were signiﬁcantly larger than that in the ADHD group in the inferior prefrontal cortex, especially in the inferior lateral prefrontal cortex bilaterally |
| Mazaheri et al. (2010) | ADHD, Control, age 8-12 years | ADHD (N=14), Control (N=11) | EEG | Cross-modal attention task | In developing children, the alpha activity was diﬀerentially modulated by the two cues and anticorrelated with midfrontal theta activity that is manifested as a functional disconnection between frontal and occipital cortex. |
| Cao et al. (2008) | ADHD, age 13.4 ±1.7 years. Control, age 13.2±1 years | ADHD (N=12) (12 males), Control (N=13) (13 males) | fMRI | Cued target detection task | Children with ADHD have deﬁcits in alerting functions and these deﬁcits are related to the abnormal activities in frontal and parietal regions subserving top-down attention control processes. |
| Batty et al. (2010) | ADHD, age 12.48±1.86 years. Control, age 12.82±1.69 years | ADHD (N=25) (24 males), Control (N=24) (23 males) | fMRI, EEG | SART | Relative to controls, children with ADHD had smaller whole brain volume and lower gray matter, but not white matter, volumes in all lobes. An analysis of frontal regions showed a signiﬁcant interaction of group by region. |
| Molenberghs et al. (2009) | Ischemic stroke patients, age 34-84 years. | N=44 | fMRI | SART | Commission errors and post-error slowing in the SART mainly probe right inferior frontal integrity. |
| Kramer et al. (2008) | TBI, Control, age 9.4 years | TBI (N=5), Control (N=8) | fMRI | CPT | All children activated similar networks of brain regions relevant to sustained attention processing, but the TBI group demonstrated frontal and parietal regions of signiﬁcantly greater activation relative to controls. |
| Skau et al. (2019) | TBI-MF, Control, age 20-65 years | TBI-MF (N=20), Control (N=21) | fNIRS | Stroop-Simon test | Pathological TBI-MF have a reduced ability to recruit the frontal cortex, which is correlated with self-reported mental fatigue. |
| Epstein et al. (2009) | ADHD, Control, age 12-18 years | ADHD (N=10), Control (N=14) | fMRI | CPT | Children with ADHD appeared to require continued use of the right middle frontal gyrus during administration of testing one year apart while healthy comparison subjects did not activate this region. |

**Table S1: Studies of sustained attention in frontal cortex.**
