# Supplementary material for: A review of visual sustained attention: neural mechanisms and computational models
Source: PeerJ. 2023 Jun 13;11:e15351. doi: 10.7717/peerj.15351 (PMC10274610; doi:10.7717/peerj.15351)
Supplement: Supplemental Information 4 — PET, positron emission tomography; ACC, anterior cingulate cortex; DLPFC, dorsolateral prefrontal cortex; fMRI, functional magnetic resonance imaging; SART, sustained attention to response task; dACC, dorsal anterior cingulate; SMA, supplementary motor area; IP, inferior parietal gyrus; ADHD, attention-deficit/hyperactivity disorder; NIRS, near-infrared spectroscopy; CPT, continuous performance test; rt-fMRI, real-time fMRI; EEG, electroencephalogram; ERP, event-related potential; APOE, carriers of the Apolipoprotein E; RVIP, rapid visual information processing. [file peerj-11-15351-s004.docx]

| **Author** | **Type of subjects** | **Number of subjects** | **Research method** | **Neuropsychological tests** | **Main ﬁndings** |
| --- | --- | --- | --- | --- | --- |
| Arbizu et al. (2001) | Healthy, age 18-37 years | N=10 (7 males) | PET | Counting task | Signiﬁcant activation was seen in the ACC, DLPFC, and inferior parietal cortex during task. |
| Forster et al. (2015) | Healthy, age 21.5 ±2.3 years | N=23 (3 males) | fMRI | SART | DLPFC activity to “Go” trials, and dACC activity to “No Go” trials were associated with faster error-free performance. |
| Ojeda et al. (2002) | Schizophrenia, age 27.55±9.4 years. Control age 26.10.2±6.95 years | Schizophrenia (N=11) (10 males), Control (N=10) (7 males) | PET | Counting task | During all counting conditions, additionally to SMA, DLPCF, precentral gyrus, cingulate, cerebellum, and IPG. |
| Wang et al. (2013) | ADHD, Control, age 7-12 years | ADHD (N=28) (25 males), Control (N=31) (16 males) | fMRI | SART | There are distinct alternations in neural circuits related to sustained attention and executive control in children with ADHD. |
| Niiyama et al. (2022) | Healthy, age 20-79 years | N=18 | NIRS | CPT | Behavioral inhibition correlates with activity in the DLPFC and right-side ACC during the CPT.. |
| Rodrigues et al. (2018) | Meditator, age 45.43±8.29 years. Control age 45.35±8.14 years | Meditator (N=23) (9 males), Control (N=17) (3 males) | fMRI | Stroop word-color task | Sustained attention to repetitive stimuli during stroop word-color task is mediated by the precuneus/posterior cingulate cortex. |
| Zilverstand et al. (2017) | ADHD, age 34±11 years. Control, age 39.8±15 years | ADHD (N=7) (3 males), Control (N=6) (3 males) | fMRI | SART | Rt-fMRI neurofeedback training targeted at increasing activation levels within dACC in adults with ADHD leads to a reduction of clinical symptoms and improved cognitive functioning. |
| Bayer et al. (2018) | Healthy, age 23.4 ±3.8 years | N=15 (8 males) | EEG, fMRI | Face-word Stroop task | ERP makers of visual processing within 100ms after stimulus onset showed covariation with brain activation in precuneus, posterior cingulate gyrus, left middle temporal gyrus, left IFG and frontal operculum, and a left lateral parietal-occipital cluster. |
| Franzmeier et al. (2018) | APOE -ε4 allele, age 21.04±2.64 years. Control age 21.00±2.00 years | Carriers (N=27) (9 males), Control (N=26) (12 males) | fMRI | RVIP | Signiﬁcantly greater activity was observed in regions including frontal, cingulate, inferior parietal, visual cortex and cerebellum. |
| Suskauer et al. (2008) | ADHD, age 10.8 ±1.2 years. Control age 10.8±1.3 years | ADHD (N=25) (15 males), Control (N=25) (15 males) | fMRI | SART | Children with ADHD showed decreased activation in the inferior frontal gyrus, and ACC, right middle/superior frontal gyrus, and bilateral rostral pre-SMA. |

**Table S2: Studies of sustained attention in cingulate cortex.**
